# Protocol-Dependent Effects on Colloidal Characterization and Drug Loading/Release Analysis of Thermosensitive PNIPAM-co-COOH Microgels

**DOI:** 10.3390/gels12070628

**Published:** 2026-07-14

**Authors:** José López-Molina, Alba Garrido-Rodríguez, María Tirado-Miranda, Delfi Bastos-González, Miguel A. Fernández-Rodríguez, Carmen Casas-Herce, Adri Escañuela-Copado, Arturo Moncho-Jordá, Irene Adroher-Benítez, J. Manuel López-Romero, Ana B. Jódar-Reyes, José M. Peula-García

**Affiliations:** 1 Biocolloid and Fluid Physics Group, Applied Physics Department, University of Granada, Granada 18071, Spain; josel@ugr.es (J.L.-M.); albagaro1989@gmail.com (A.G.-R.); mtirado@ugr.es (M.T.-M.); dbastos@ugr.es (D.B.-G.); mafernandez@ugr.es (M.A.F.-R.); ccasash@hotmail.com (C.C.-H.); escanuela@ugr.es (A.E.-C.); moncho@ugr.es (A.M.-J.); iadroher@ugr.es (I.A.-B.); 2 Department of Organic Chemistry, Faculty of Sciences, University of Málaga, 29071 Málaga, Spain; jmromero@uma.es; 3 Department of Applied Physics II, University of Málaga, 29071 Málaga, Spain

**Keywords:** PNIPAM thermosensitive microgel, colloidal characterization, drug encapsulation, drug release, convection-corrected DLS, static light scattering, dialysis

## Abstract

This work analyzes protocol-dependent effects on the colloidal characterization and drug loading/release analysis of model thermosensitive PNIPAM-co-COOH microgels and shows how they can be quantified or minimized through targeted methodological refinements. Findings reveal that standard single-beam DLS underestimates the collapsed hydrodynamic radius by 18% at 43 °C due to thermal convection. After drift correction, 3D-DLS combined with SLS provides a consistent description of thermally induced collapse, pH-dependent swelling and core–corona structure. Regarding drug delivery, loading efficiency for Doxorubicin and 5-Fluorouracil is maximized near the volume phase transition temperature, where hydrophobic interactions are strongest. For release studies, dialysis is recommended, but free-drug blanks are required to account for membrane-induced delay and ensure accurate early kinetic profiles. By integrating TEM, AFM, SLS, DLS, NTA and LDE, this study establishes a robust framework for the colloidal characterization of thermosensitive microgels. These refinements reduce experimental bias and may be extended to related soft nanocarriers.

## 1. Introduction

Poly(N-isopropylacrylamide) (PNIPAM) microgels are thermosensitive nanoparticles (NPs) that have long been studied for their potential application as drug delivery systems [1,2,3,4]. Hydration of the polymer chains promotes local ordering of water molecules around the amide groups through hydrogen bonding. As temperature rises, enhanced molecular motion disrupts these hydrogen bonds, leading to a loss of the local order of the water around the polymer. This disruption favors hydrophobic attractions among the isopropyl groups. A critical temperature, known as the volume phase transition temperature (VPTT), marks this behavior: below the VPTT, microgel particles are swollen due to hydration, whereas above it, they collapse as hydrophobic interactions dominate [5].

The VPTT for PNIPAM in water is around 32 °C. These microgels can be modified with a wide variety of polymers increasing the transition temperature to 37 °C and modulating the hydrophobicity/hydrophilicity to enhance the loading of drug molecules with different hydrophobicity [4]. Thus, different drug molecules can be efficiently loaded into the copolymeric microgel system below its VPTT and subsequently released at a specific target site by gently raising the temperature above that VPTT. Furthermore, copolymerization of NIPAM with acrylic or butenoic acids preserves the thermosensitive behavior of PNIPAM without altering its VPTT and introduces carboxylic functionalization to achieve two main goals: a dual response to external temperature and pH changes, and the capability to covalently attach different targeting agents [6,7,8]. The synthesis and thermosensitive behavior of the PNIPAM-co-COOH formulation used in this work, including its VPTT around 32 °C, have been previously reported in reference [7]. In the present work, temperatures below and above this known VPTT were selected to analyze protocol-dependent characterization, drug loading and release effects.

Once the nanoparticle synthesis process has been controlled, determining their colloidal properties—such as morphology, hydrodynamic size, and charge under various physiological conditions, alongside their stability—becomes essential. Intrinsic properties of microgels, such as their low optical contrast with the medium in which they are normally dispersed (aqueous medium) together with their response to temperature changes introduce difficulties in their colloidal characterization using standard techniques. For instance, in colloidal systems, the application of temperature variations significantly influences light scattering measurements, particularly when dynamic light scattering (DLS) is used to probe particle dynamics. Temperature variations in the system can induce thermal convection, which impacts the scattering measurements by introducing convective currents. These currents result in a directed motion of the particles, leading to a drift velocity superimposed on the random Brownian motion. When thermal gradients are present, the convective flow of the fluid causes particles to move systematically, creating an additional velocity component that can alter the intensity autocorrelation function typically used in DLS analysis. We modeled this phenomenon in a previous work using a 3D-DLS setup [9] and found that the drift velocity driven by convection manifested as oscillations in the autocorrelation function, complicating the interpretation of DLS data. In addition, a faster decay of the correlation function arose because particles were carried out of the scattering volume more quickly than diffusion alone would predict. This effect can be particularly pronounced in systems where the thermal gradient is steep, leading to stronger convective currents.

When residual thermal gradients persist during DLS measurements, the apparent diffusion coefficient may contain both Brownian and convective contributions. Reaching a stable set temperature at the monitored scattering volume does not necessarily ensure thermal uniformity throughout the entire sample cell, and the convective contribution may therefore remain unnoticed. Neglecting this effect can lead to errors in the calculated diffusion coefficient and an underestimation of the particle diameter.

Regarding the applicability of microgel nanoparticles, effective drug loading and controlled drug release are essential requirements when developing micro- or nano-delivery systems. To this end, it is critical to study the loading and release patterns of drugs with different physicochemical characteristics. Thus, the permeability of microgels and the diffusive processes of these drug molecules throughout the polymeric network of microgels are determinants, especially considering the different situations in which the systems present swollen or collapsed states depending on temperature and/or other physicochemical conditions [4,10,11].

PNIPAM-based microgels exhibit a marked hydrophilic character in their polymeric core, making the incorporation and delivery of hydrophobic molecules highly challenging [2,12,13]. Despite this limitation several studies have utilized these systems to encapsulate hydrophobic drugs, i.e., paclitaxel or ibuprofen [4,14,15]. However, the use of hydrophilic drugs has been widely described, and these types of active drugs may be good candidates to study the encapsulation processes in the polymeric matrix of the designed microgel and the subsequent delivery through diffusive processes from its mesh to the bulk solution, below (swollen state) and above (de-swollen state) its VPTT. For instance, Doxorubicin (DOX) and 5-Fluorouracil (5FU) are classified as BCS (Biopharmaceutics Classification System) III hydrophilic drugs. They are widely used; DOX is particularly used in cases of leukemia, lymphoma, lung, ovarian and breast cancer, whilst 5FU is employed in the treatment of stomach, colon and breast cancer. Both are soluble in water (hydrochloride DOX, 10 mg/mL and 5FU, 12.2 mg/mL). However, while DOX is a basic drug with a positive charge at physiological pH (the pKa of Doxorubicin is 8.2) [16], 5FU is a good model of non-ionized drug in physiological media [17]. An additional advantage of these drugs is the possibility of a simple quantification process using spectrophotometric procedures and the corresponding standard concentration curves.

Most encapsulation processes reported in the literature for 5FU involve physical adsorption and/or diffusion of drug molecules inside microgel nano- or micro-particles after simple addition of different amounts of drug to an aqueous solution of microgel NPs using stirring at room temperature (swollen state) [7,18,19,20,21]. Similarly, DOX was encapsulated inside several hybrid PNIPAM nanogels by drug diffusion into the mesh of the nanoparticles, where electrostatic interactions and hydrogen bonding between the drug molecules and the gel matrix take place [22,23]. However, different parameters could affect the drug encapsulation process in PNIPAM microgels and they should be optimized, for instance, the drug/polymer ratio, drug/polymer interactions and encapsulation temperature. Even the method used to quantify the amount of encapsulated drug, with centrifugation and analysis of the supernatant being the most used strategy, can also lead to erroneous results.

Once the drug molecules are trapped inside the microgel mesh, the diffusive processes of these encapsulated molecules will be decisive for its subsequent delivery from the matrix to the dispersion medium. For thermosensitive PNIPAM microgels, this drug release mechanism will be very dependent on the VPTT. A gradual release would be expected below the VPTT, while the shrinkage of the microgel mesh above the VPTT would produce a rapid diffusion and release of the drug molecules [24].

Accordingly, designing an appropriate protocol to conduct release experiments is essential to accurately analyze drug delivery kinetics without interfering with the release process itself. In this regard, dialysis is the most suitable technique, as it reproduces adequate conditions, whether the microgel particles are in the swollen or collapsed state, while allowing control over key parameters such as pH and ionic strength of the elution medium, as demonstrated in numerous procedures reported in the literature [4,7,18,19,21,22,23]. However, the permeation process of the drug molecules through the dialysis membrane pores could also play an important role in the correct interpretation of experimental results. In fact, the drug release process into the bulk external solution is the sequential combination of two processes: firstly, the drug is released from microgel particles into the dispersion medium inside the dialysis bag and, next, the drug permeates across the dialysis membrane. Thus, dialysis experimental data are not necessarily descriptive of the real kinetic drug release from the colloidal system if several processes such as drug–membrane interactions, drug molecular aggregations and/or drug-microgel partitioning (equilibrium distribution constant) are not considered [25]. Most dialysis drug release experiments from PNIPAM microgels described in the literature ignore this membrane effect. However, in other types of nanoparticles, the membrane effect is noted [26], and the quantification of a membrane permeation rate constant to reach the normalization of the release profiles was proposed [25,27]. Ultracentrifugation is an alternative method also proposed in the literature [28], but the applied external force must alter the drug diffusion behavior, particularly for drugs trapped in the highly hydrated matrix of microgels [29].

The aim of this work is to examine how commonly used characterization, drug loading and release protocols perform when applied to a thermosensitive PNIPAM-co-COOH microgel system. Rather than proposing a universal standardization framework, we provide a quantitative analysis of the experimental factors that condition the interpretation of size, morphology, electrokinetic behavior, encapsulation efficiency and release kinetics in this model system. Emphasis is placed on distinguishing intrinsic microgel behavior, such as temperature- and pH-dependent swelling, from protocol-dependent effects, such as convection-driven decorrelation in DLS, analysis-parameter sensitivity in NTA, sample preparation effects in microscopy, drug loss during loading quantification and membrane-induced delay in dialysis release experiments. On this basis, we derive practical considerations that may also be useful for interpreting characterization and drug delivery data in related soft thermosensitive microgels, provided that differences in particle size, optical contrast, polydispersity, medium composition and thermal-control geometry are taken into account.

In the first step, we carried out an in-depth colloidal characterization of the microgel system synthesized according to the methods reported by [6,7]. Specifically, the NIPAM monomer was polymerized with 3-butenoic acid to obtain PNIPAM-co-COOH NPs with a charge dependent on the pH of the medium. The colloidal characterization covered the analysis of morphology (by transmission electron microscopy (TEM), atomic force microscopy (AFM), and static light scattering (SLS)), hydrodynamic size (via DLS and nanoparticle tracking analysis (NTA)), and electrokinetic behavior through Laser Doppler Electrophoresis (LDE). The colloidal stability was studied under different conditions, such as variations in pH and temperature of the dispersion medium. The existence of a temperature gradient in the hydrodynamic size measurement cell was also analyzed.

Once the microgel colloidal system was fully characterized, the ability of the nanoparticles to encapsulate and release different drug molecules was studied. This included the analysis of different parameters affecting the drug encapsulation process, that is, the drug/polymer ratio, drug/polymer interactions and encapsulation temperature, with special attention to the VPTT. DOX and 5FU were the selected drugs, with hydrophilic character but different charge at our working conditions: DOX has positive charge and 5FU is neutral in physiological media.

Finally, different drug release characterization protocols were reviewed, and their advantages and disadvantages were pointed out. We describe the kinetics of release of both drugs under different conditions with a dynamic dialysis methodology under magnetic stirring of the external solution and using a conventional membrane with the typical molecular weight cut-off MWCO of 12 kDa, which is widely referred to in the literature. We worked at a physiological temperature slightly above the VPTT of the hydrogel. Considering the problems previously outlined for this technique [25,27,30], we analyzed the permeation of both drugs through the dialysis membrane working with different samples, free and encapsulated drugs, and different inner and outer volumes around this membrane. This was carried out to adequately overcome the challenges of this technique and correctly reflect the drug release kinetics of these microgels.

Although this work focuses on PNIPAM-based microgels and the experimental setups described above, the experimental hurdles analyzed here are not exclusive to this system. However, their magnitude is expected to depend on: (i) the particle characteristics (size, optical contrast and polydispersity); (ii) the temperature gradient in the measurement cell; and (iii) the composition of the medium (pH, ionic strength, and biological components). Therefore, when these recommendations are applied to other thermosensitive microgels or drug-loaded soft nanoparticles, it is advisable to explicitly report these parameters and, when necessary, re-optimize the corresponding experimental protocol.

## 2. Results and Discussion

### 2.1. Characterization of the Microgels

Following the synthesis and purification of the microgel nanoparticles, it is necessary to know their main colloidal properties, i.e., morphology, hydrodynamic size, charge under different physiological conditions of the medium, and stability under these conditions. The special characteristics of microgels, such as their softness due to their internal composition formed by a polymer network and water, and their response to temperature changes, make it difficult to apply standard techniques. Therefore, in this work, different techniques are applied to obtain some of these properties, and their advantages and disadvantages are analyzed with the aim of optimizing the characterization process.

#### 2.1.1. Particle Morphology

Imaging techniques such as TEM or AFM are usually the first choice to get direct images of the nanoparticles to obtain information on their shape, but also on their size and polydispersity. Both techniques present the problem that the preparation of the sample required for measurement may alter the structure that the particles would have when dispersed in an aqueous medium. This is particularly important in microgels with high softness. An alternative would be SLS, which, although it does not allow direct observation of the shape, does allow to measure averaged information in an aqueous medium through the form factor (P(q)) and the geometric radius. In addition, we will present below how the core–shell structure of this type of microgel can be characterized using both AFM and SLS.

**Figure 1:**
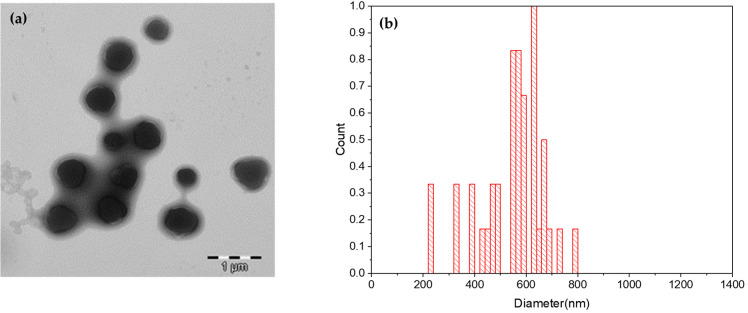
(**a**) TEM micrographs of microgel particles. Scale bar: 1 µm; (**b**) normalized size distribution (histogram) obtained from TEM micrographs.

Figure 1a shows a micrograph of the microgel NPs, confirming their spherical shape. A change in electronic density in their structure can also be seen, with a dark center and a halo around it. In principle, the drying conditions would cause the particles to collapse, so this halo would not correspond to the microgel shell. This halo is also seen in images of other microgels [10] and is an artifact of the sample preparation process. Regarding the size, in Figure 1a we observed microgel nanoparticles with a rather polydisperse size distribution. From the analysis of more than 100 NPs we obtained diameters between 220 and 790 nm. The mean diameter was (540 ± 130) nm, and the mode was 625 nm (see histogram in Figure 1b). Only the dark part of the microgels was considered when calculating the diameter. The sample was dried at 37 °C, which is above the VPTT.

**Figure 2:**
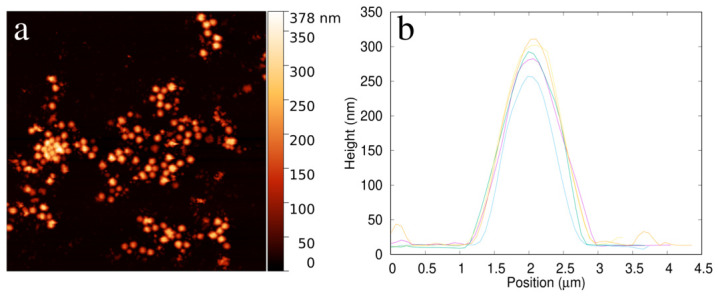
(**a**) 40 × 40 µm^2^ AFM image of the Langmuir–Blodgett microgel deposition at 5 mN/m on a silicon substrate. (**b**) Profile of 5 microgels from the AFM image.

Another standard technique to analyze the morphology of the microgels is the combination of Langmuir–Blodgett deposition and atomic force microscopy. It involves depositing the NPs at a water/air interface thanks to a spreading agent such as isopropanol and depositing the Langmuir–Blodgett monolayer at a fixed surface pressure of π = 5 mN/m onto a silicon substrate [31]. Microgels synthesized by precipitation polymerization develop a core–shell morphology, which is enhanced when a microgel is deposited at the water/air interface. This phenomenon arises from the stretching of the portion adsorbed at the interface due to the surface tension, which is only counter-balanced by the elasticity of the cross-linked polymeric network. Thus, the more cross-linked they are, the less they expand at the interface. The portion immersed in the water subphase is well dispersed in water and the portion protruding into air is collapsed [32]. From the Langmuir–Blodgett deposition at 5 mN/m and AFM characterization (Figure 2a) we can confirm the spherical shape of the microgels. The fact that there is not a full microgel monolayer despite being at 5 mN/m points to interfacial active species in the microgel dispersion that occupy the interface around the microgels. By selecting five microgels and obtaining their profiles (Figure 2b), we can observe the Gaussian profile due to the core–shell nature of the microgels. This approach allowed us to obtain profiles of individual microgels (Figure 2b).

Gaussian fits were then applied to determine the diameter d_AFM_ = 1840 ± 150 nm, height H_AFM_ = 289 ± 21 nm, and standard deviation σ_AFM_ = 380 ± 30 nm of the deposited microgels, providing evidence of their core–shell structure. In fact, for microgels without a hard core, the boundary or extent of the core within the core–shell morphology is inherently undefined; thus, the value of 2σ_AFM_ = 760 ± 60 nm serves as a good representation of such a core. Indeed, this value is in reasonable agreement with the size obtained from TEM. Nevertheless, AFM profiles are more sensitive to the microgel morphology, finding a d_AFM_ larger than the size found by TEM.

**Figure 3:**
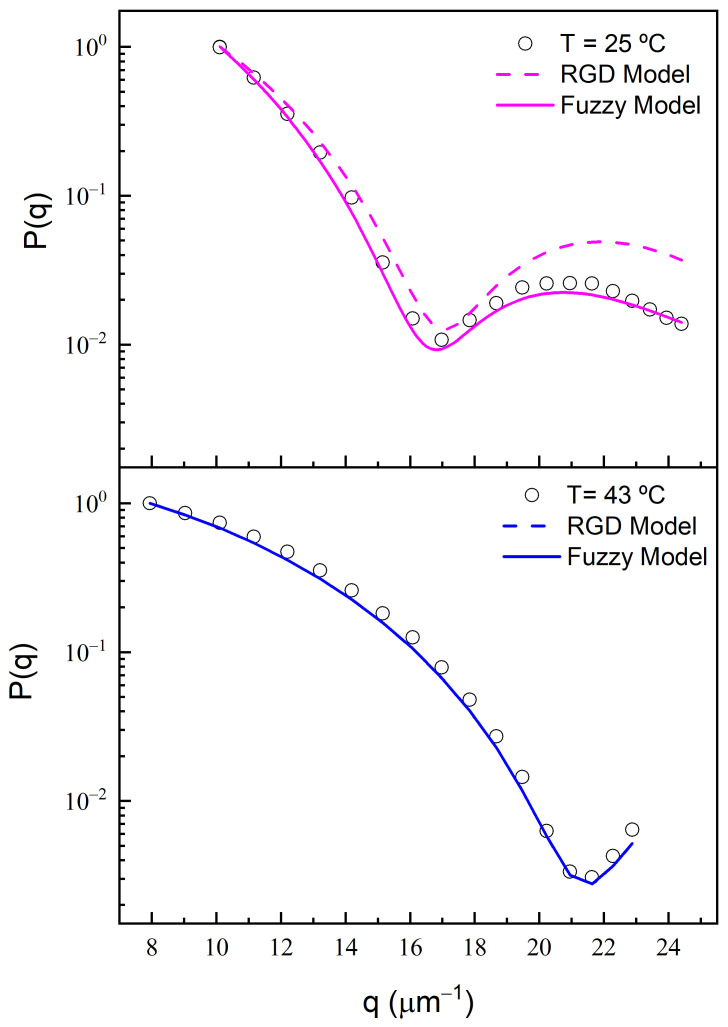
Form factor of microgels in Milli-Q water at 25 °C (**up**) and 43 °C (**down**). The solid lines correspond to the RGD model and the dash lines to the fuzzy-sphere model.

We next used SLS to probe the dimensions and diffuse corona of microgels. Figure 3 shows the static form factors measured at 25 °C and 43 °C together with fits to the Rayleigh–Gans–Debye (RGD) model and its fuzzy-sphere extension. Both form-factor curves (swollen and collapsed) exhibit a well-defined minimum whose position defines the particle radius. At 25 °C, the RGD model fails to capture the high-q behavior, whereas the fuzzy-sphere fit yields a radius R = 267 nm with a smearing parameter σ_fuzzy_ = 56 nm, corresponding to an effective static radius R_SLS_ = R + 2σ_fuzzy_ = 379 nm and revealing a swollen diffuse shell. Upon heating to 43 °C, both models converge to R_RGD_ = R ≈ 209 nm with σ_fuzzy_ ≈ 0, indicating that the corona has collapsed and the particle behaves as a nearly homogeneous sphere. These values are summarized in Table 1. SLS sensitively reports temperature-induced radius changes and quantifies the microgel shell thickness, confirming complete deswelling above the VPTT. In Section 2.1.2, we will compare the geometric and hydrodynamic radius to obtain more information about the core–shell structure in our system.

In summary, regarding the characterization of the morphology and structure of the PNIPAM microgel: (i) image techniques such as TEM and AFM can be used to obtain information about the shape and polydispersity of the NPs; (ii) AFM also provides qualitative information about the core–shell structure, which may be useful, for instance, when comparing the core–shell dimensions under different synthesis strategies; (iii) although it only provides ensemble-averaged information, SLS allows a quantitative characterization of the core–shell structure of microgels; and (iv) the dimensions obtained from TEM, AFM and SLS should not be interpreted as directly equivalent because each technique probes a different aspect of the microgel structure. TEM provides particle dimensions in the dried state, AFM reflects the morphology of microgels after interfacial deposition and spreading and SLS yields an ensemble-averaged static description of the particles in dispersion through the form factor. Accordingly, comparisons between TEM, AFM and SLS are most meaningful when used to identify consistent structural trends, such as particle shape, polydispersity or core–corona organization, rather than to establish a strict one-to-one numerical equivalence between sizes.

#### 2.1.2. Hydrodynamic Size

To measure the hydrodynamic size of a soft colloidal system, the use of complementary techniques such as NTA and DLS is highly recommended. Although both are based on the analysis of the Brownian motion of the particles in the medium, NTA allows us to obtain direct information on the polydispersity of the sample, while DLS provides us with an average size with less uncertainty. This section presents the results of this complementary study conducted on our PNIPAM-co-COOH microgel.

Nanoparticle tracking analysis is especially indicated for systems that in imaging techniques, such as TEM, show a certain degree of polydispersity, as each particle is studied separately but simultaneously, and the size distribution (i.e., concentration of particles versus diameter) is provided [33]. Correct tracking of the nanoparticle implies that there is an optical contrast between the particle and the medium in which it is suspended. From the size distribution, the mean diameter was calculated to compare with DLS results. In addition, we obtained the mode of the distribution to compare with the TEM results.

Initially, to perform valid comparative studies concerning the effect of the temperature of the medium, we fixed a sonication time of 30 min to prepare the sample from the stock. As shown later, the optimal sonication time for our system is 2 h and this was used for all hydrodynamic size measurements. Importantly, the effect of temperature observed in the initial experiments remained unchanged when the sonication time was increased, indicating that this trend was independent of sonication time.

**Figure 4:**
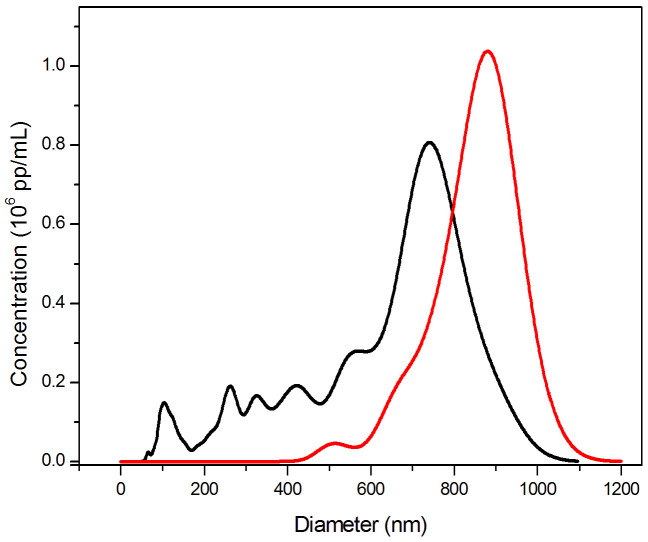
NTA size distribution of the microgel in Milli-Q water at T = 37 °C (black line) and T = 43 °C (red line). Sonication time: 30 min. CL = 14 and DT = 7.

When the microgel was in a swollen state (T < VPTT), NTA did not provide reliable analysis, since the refractive index of the particle was very similar to that of the aqueous medium and the software could not correctly track the NPs. Even when the temperature was around the VPTT (32 °C), the analysis was complicated, since the number of completed tracks was well below that corresponding to a correct statistical analysis and reproducible size distributions could not be obtained. Only for temperatures of 37 °C and 43 °C, the software provided us with size results. Figure 4 shows the distributions obtained for temperatures of 37 °C (body temperature) and 43 °C (well above VPTT). The same capture and analysis parameters (Camera Level, CL = 14, and Detection Threshold, DT = 7) were used in both cases to ensure that the two distributions were compared under identical NTA acquisition and processing conditions.

The size distribution corresponding to 37 °C indicates higher polydispersity than expected, with the presence of small particles not observed in TEM. At this temperature, the contrast is still insufficient to obtain acceptable results with this technique. At 43 °C, although the width of the size distribution is considerably reduced, the peak shifts to higher values, which does not correspond to the expected collapse. Specifically, the mean and mode values of the diameter were (850 ± 90) nm and 880 nm. To check if the problem was that the sample needed more sonication time, we compared the results obtained after sonicating for 0.5, 1, 2 and 3 h and then measuring at 43 °C. We observed that the size was sonication time-dependent but it reached a constant value after 2 h of sonication, and in these conditions, the number of valid tracks was the highest, so it was set as the optimum time for the sample preparation. The mean and mode values of the diameter were (730 ± 100) nm and 730 nm, respectively.

Finally, we highlight the effect of the DT parameter on the NTA results. When we repeated the analysis of the sample at 43 °C using DT = 5, we obtained a mean diameter of (480 ± 100) nm and a mode of 495 nm. This means that the choice of this parameter has an important effect on the results obtained from NTA for this system. A higher DT (as DT = 7, for instance) gives more weight to larger particles, as they shine brighter than smaller particles composed of the same material. While this dependence is usually less critical in hard particle systems, it becomes particularly important in soft particle systems such as the one studied here. In this case, the NTA size distribution is highly sensitive to the analysis parameter. Accordingly, the influence of DT should be assessed and reported, and the same DT should be used when comparing samples within a given experimental series.

With the standard single-beam DLS instruments and measurement configurations available in this study, the software did not return reliable size estimates over the temperature range examined. The low data quality was mainly attributed to sample polydispersity in both the swollen and collapsed states. Use of a capillary cell, which is suitable for large microgels and reduces the sample volume, improved the measurements in some cases (shown in Appendix A, Table A1), although the instrument still indicated that cumulant analysis was not suitable for this sample. In the collapsed state, the cumulant analysis yields a diameter around 1700 nm and a PDI of 0.7. From the analysis of the size distribution by intensity of the measurements of good quality in Milli-Q water, diameters in the collapsed state were (970 ± 200) nm at 42 °C.

The 3D-DLS setup allows for a more controlled analysis of the intensity autocorrelation function than the conventional DLS device. We therefore used it to obtain reliable hydrodynamic radii for the microgels.

**Figure 5:**
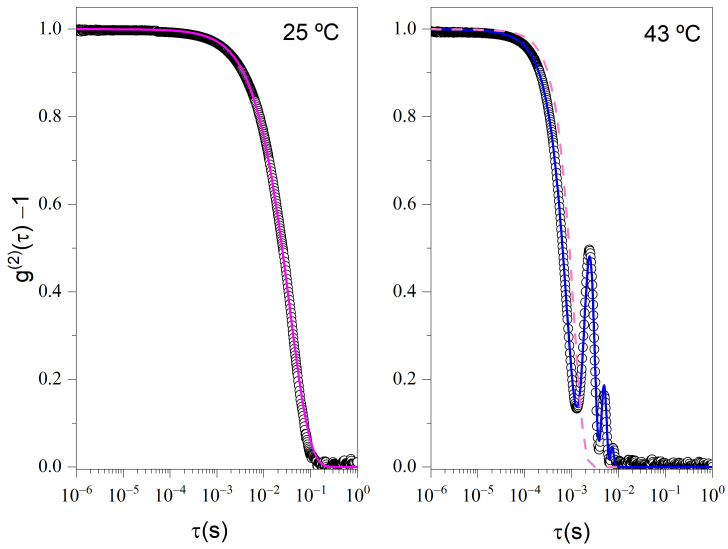
Intensity correlation function of microgels in Milli-Q water at 25 °C (**left**) and 43 °C (**right**). The dash line corresponds to the cumulant model, and the solid line represents the convection-corrected model. Both lines overlap at 25 °C.

Figure 5 shows the normalized intensity correlation functions measured in Milli-Q water at 25 °C and 43 °C. At 25 °C, the correlation decays exponentially, as expected for Brownian diffusion. At 43 °C, an initial exponential decay at short delay times is followed by pronounced oscillations at longer delays. It has recently been demonstrated that such oscillations arise from particle drift driven by convective flow induced by the thermal gradient between the 43 °C scattering volume and the ambient-temperature headspace. The dashed and solid lines in Figure 5 correspond to fits using the cumulants method (Equation (4), yielding R_h_^cum^) and the convection-corrected model (Equation (5), yielding R_h_^conv^). The extracted radii are collected in Table 1. At 25 °C, both approaches agree within ≈2%, indicating negligible convective artifacts. At 43 °C, however, the difference grows to approximately 18%, and only the drift-corrected model accurately reproduces the oscillatory tail. Heating past the volume phase transition yields a sharp collapse of the microgels, reducing R_h_^conv^ from 530 nm to 265 nm. At 43 °C, the oscillatory tail revealed residual convective drift associated with a vertical temperature gradient across the cell. Including this contribution in the analysis was therefore required to obtain the hydrodynamic radius and confirm the thermally induced collapse of the microgel.

Oscillatory or otherwise anomalous intensity autocorrelation functions have been previously reported in DLS measurements of absorbing samples and systems subjected to external flow or thermal gradients. Moulin et al. related long-time oscillations in light-absorbing supramolecular polymer solutions to laser-induced thermogravitational convection [34]. Earlier studies reported oscillatory or non-Brownian correlation functions in cross-beam electrophoretic light scattering and absorbing polymer solutions [35,36] whereas Katayama et al. found flow-dependent changes in the apparent diffusion of nanoparticles [37]. These observations show that deviations from purely Brownian decorrelation are not specific to the present microgel although its effect on hydrodynamic sizing of thermosensitive microgels has received less attention.

In standard single-beam homodyne DLS instruments, where the incident beam and detector define a single scattering vector in the scattering plane, convective drift along the vertical direction of the cell does not necessarily produce an oscillatory contribution in the measured intensity autocorrelation function. As a result, the correlation function may retain an apparently normal diffusive decay even when convection contributes to the loss of correlation and biases the apparent diffusion coefficient. In contrast, the two-beam 3D-DLS geometry used here is sensitive to this drift contribution because the difference between the two scattering vectors has a vertical component. Therefore, the drift velocity can be accessed through the oscillation frequency, ω = Δq·v, allowing the Brownian and convective contributions to be separated [38]. This issue is particularly relevant in routine measurements performed substantially above room temperature because reaching a stable set temperature at the scattering volume does not necessarily ensure thermal uniformity throughout the whole sample cell. Residual vertical temperature gradients may persist in tall cells, large sample volumes, partially thermostated geometries, or configurations in which the headspace or part of the sample remains exposed to ambient temperature.

This effect can be minimized in routine measurements by reducing the sample volume, using capillary cells when compatible with the instrument, avoiding partially thermostated sample volumes and ensuring homogeneous thermal equilibration of the whole cell. When convective drift is detected or cannot be ruled out, its contribution should be measured and included in the analysis. The discrepancy observed above the VPTT therefore arises from applying a purely diffusive model to data affected by both Brownian motion and residual convective drift.

Finally, it can be noted that at T = 43 °C the convection corrected diameter (2·R_h_^conv^ = 2 (265 ± 19 nm) = 530 ± 38) is in good agreement with the mode value (495 nm) obtained by NTA at DT = 5. The agreement between both techniques supports the reliability of the collapsed-state size obtained for the microgel and reinforces the interpretation that the temperature increase above the VPTT leads to particle collapse rather than to an apparent size decrease caused only by analysis artifacts.

Transient interparticle association may occur during heating because dehydration of the PNIPAM-rich network above the VPTT increases the relative importance of hydrophobic interactions [5]. Under the dilute light scattering conditions used here, however, there is no indication that this association dominates the measured size. At 43 °C, the SLS form factor retains a well-defined minimum and is fitted by an almost homogeneous collapsed sphere within the fuzzy-sphere description of thermosensitive microgels [39,40], while R_h_^conv^ decreases strongly relative to the swollen state and is consistent with the NTA mode. The combined data therefore point to single-particle deswelling, with convective decorrelation accounting for the difference between the cumulant and drift-corrected DLS radii. Short-lived contacts during passage through the transition may still occur, but they are not resolved by the present measurements.

To further assess the internal structure of the microgels, we compared the static radii obtained from SLS with the hydrodynamic radii from 3D-DLS. For the fuzzy-sphere fits, we consider R_SLS_ = R + 2σ_fuzzy_ as an effective static radius and R_h_^conv^ as a dynamic radius (Table 1). The resulting static-to hydrodynamic size ratios are R_SLS_/R_h_^conv^ ≈ 0.72 (at 25 °C) and R_SLS_/R_h_^conv^ ≈ 0.79 (at 43 °C). The larger ratio in the collapsed state indicates that the particle behaves more like a compact object at high temperature, whereas the smaller ratio in the swollen state reflects the presence of a soft, extended corona and the permeable nature of the network at low temperature.

Physically, R_h_^conv^ exceeds R_SLS_ because hydrodynamic interactions are sensitive not only to the denser core but also to the dilute outer corona and the solvent dragged along with the particle. In SLS, the scattered intensity is weighted by the local polymer concentration, so regions with very low polymer density in the outer corona contribute only weakly to I(q). As a result, the fitted static radius R_SLS_ is mainly determined by the dense core and the inner part of the corona, while the most diffuse tails are almost “invisible” to scattering. In contrast, DLS does not probe polymer density but hydrodynamic friction: any polymer segment that moves coherently with the microgel (core plus fuzzy shell), together with the hydration layer and part of the solvent trapped in the network contributes to the drag. This additional friction effectively extends the hydrodynamic boundary of the particle beyond R_SLS_, leading to R_h_c^onv^ > R_SLS_ for soft microgels with a diffuse corona.

Within the fuzzy-sphere model, the effective static radius R_SLS_ is slightly larger than the radius of gyration R_g_, but both quantities are strongly correlated and capture the overall extension of the microgels. We therefore used the ratio R_SLS_/R_h_^conv^ as an effective static-to-hydrodynamic size ratio and compared its magnitude with the R_g_/R_h_ values reported for soft colloids in the literature. Reported R_g_/R_h_ ratios for soft colloids typically lie in the range of 0.60–0.80 [41,42], with values around 0.68 for slightly cross-linked PNIPAM microgels and down to ≈0.60 for protein nanogels and collapsed PNIPAM microgels with higher cross-linker content [39]. Although R_SLS_ slightly overestimates the true R_g_, our effective static-to hydrodynamic ratios R_SLS_/R_h_^conv^ ≈ 0.72 (at 25 °C) and R_SLS_/R_h_^conv^ ≈ 0.79 (at 43 °C) fall within this range and exhibit the expected trend of a lower ratio in the swollen state and a higher ratio in the collapsed state. This behavior is fully consistent with a dense-core/soft-shell microgel architecture at low temperature that transforms into an almost homogeneous sphere above the VPTT, in agreement with the shell thickness and effective radius obtained from SLS, the collapse inferred from the temperature dependence of R_h_^conv^, and the core–shell morphology observed by AFM.

In summary, regarding the characterization of the hydrodynamic size of the PNIPAM microgel, (i) NTA was informative mainly in the collapsed state, where the optical contrast permitted reliable tracking; (ii) NTA distributions were sensitive to CL and DT, so acquisition and analysis settings must be established from tracking quality and kept fixed within each comparative series; (iii) the standard single-beam DLS configurations used here did not yield reliable size estimates for this system, even in the collapsed state; (iv) the 3D-DLS device provided the hydrodynamic radius in both swollen and collapsed states; at 43 °C, the oscillatory tail revealed a residual convective contribution that had to be included in the analysis; (v) the ratio between geometric and hydrodynamic radii was used to validate the core–shell picture; and (vi) the magnitude of convective artifacts in DLS and the practical detectability limits of NTA depend on the instrument geometry and temperature-control strategy (thermal gradient in the measurement cell) as well as on particle size/optical contrast and polydispersity; thus, these aspects proved important and should be checked and explicitly reported for systems studied under comparable conditions.

#### 2.1.3. Electrokinetic Behavior

**Figure 6:**
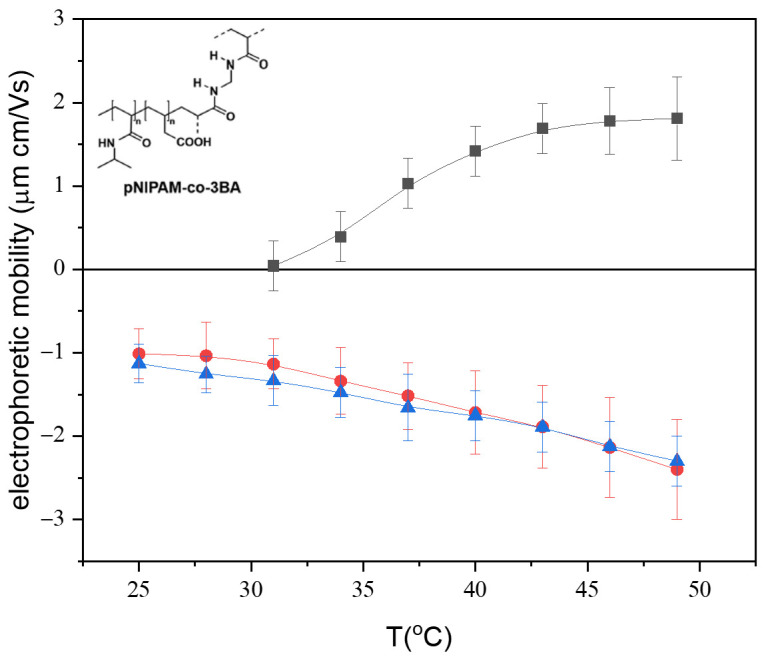
Electrophoretic mobility of microgel versus medium temperature at different pHs: pH 4 (squares), pH 7 (circles), pH 9 (triangles).

To characterize the electrokinetic behavior of a thermosensitive microgel, measuring the electrophoretic mobility as a function of temperature proved informative (Figure 6). In addition, because some microgel components contain pH-dependent charged groups, measurements at different pH values also proved necessary in the present system. The co-monomer 3-butenoic acid (3BA) contains carboxyl groups, which confer negative charge under basic pHs. Furthermore, the initiator V50 contains amino groups, which are positively charged under acidic conditions.

Considering that the VPTT of the microgel is around 32 °C, measurements were performed from room temperature (swollen state) to a temperature that ensured the collapsed state (49 °C) (Figure 6). The mobility was strongly dependent on pH. At acidic pH (pH 4), the device did not provide any quality results below T = 31 °C. At that temperature (slightly below VPTT), the mobility was around zero. At T > VPTT, it changed to positive values, indicating that the amino groups are positively charged while the carboxyl groups are in their neutral form. The values obtained at pH 7 and 9 were similar within the experimental error and remained negative due to the presence of negatively charged carboxyl groups, thereby confirming the amphoteric nature of the microgel. Error bars indicated that the mobility distribution was wide in all conditions. When analyzing the electrophoretic mobility of microgels as a function of temperature, it is important to move beyond classical hard-sphere models and account for their soft, electropermeable nature. Unlike rigid colloids where solvent flow is deflected at a sharply defined slipping plane, a swollen PNIPAM microgel allows the surrounding aqueous medium to penetrate and flow through its porous polymer matrix. Consequently, its electrophoretic behavior is governed by a dynamic balance between the electrical driving force acting on the internal fixed charges and the hydrodynamic friction. During the temperature-induced volume phase transition, the drop in mobility cannot be attributed solely to shifting charge density; it is heavily driven by the structural transformation from a highly drained, permeable network into an impenetrable, rigid-like sphere, which fundamentally alters the hydrodynamic drag and shifts the effective shearing boundary [43,44]. In Figure 6 we observe how the mobility of the microgel increases (in absolute value) as the temperature increases. At pH 4 this increment in mobility is sharper as the VPTT is surpassed, but for all pHs the mobility is maximum in the collapsed state due to the relocation of the charged groups in the external hydrophilic environment of the collapsed microgel [10].

In summary, to correctly characterize the electrokinetic behavior of a thermosensitive microgel: (i) the effect of temperature on electrophoretic mobility must be assessed using values both below and above the VPTT; (ii) the effect of the medium pH should be evaluated, especially when pH-dependent charged groups are present; and (iii) calculating the standard deviation from the electrophoretic mobility distribution is essential to discuss differences between different experimental conditions.

#### 2.1.4. Colloidal Stability in Different Media

Once it was established that the pH of the medium affects the electrokinetic behavior of the microgel, studying the effect of the medium characteristics on colloidal stability became relevant for the present system, particularly when considering storage conditions or subsequent use in physiological media. It should be noted, however, that the stability data reported here in controlled aqueous/buffer media should not be interpreted as sufficient evidence for intravenous applicability, since biologically relevant media may introduce additional effects related to ionic strength, proteins and biomolecular corona formation. Thus, we evaluated whether the hydrodynamic size and static radius of the microgel in the collapsed state (43 °C) were affected by the characteristics of the dispersion medium (Milli-Q water, and buffers at pH 4 and pH 7). For this purpose, we used NTA (with CL = 14 and DT = 5), 3D-DLS and SLS.

The results from the size distribution by NTA (mean and mode values of the diameter), at the initial time (t = 0) and after 40 min are presented in Table 2. Regarding temporal stability, a shift in the size distribution towards higher values was observed only when Milli-Q water was used as the medium; however, when accounting for the experimental error of the technique, no change in the mean value was found. In the pH 4 buffer there was no significant change in the distribution over time, indicating that the particles remained stable under these conditions. In the pH 7 buffer, however, the main peak shifted toward lower values, resulting in a distribution more consistent with that observed in the same medium via TEM. Therefore, the system did not aggregate; instead, it evolved toward a more compact configuration.

The type of medium also appeared to affect the statistical values of the diameter (mean and mode), which were lower at pH 4, both at the initial time and after 40 min; however, the standard deviation (SD) was so high that a significant change in the mean cannot be confirmed. To explain these results, we compared the mobility values of the system in the different media at T= 43 °C (Table A2). They were similar in absolute value and did not change after 40 min. Then, the observed shift in the size distribution for Milli-Q water cannot be explained using these electrokinetic results. Thus, although NTA allows us to measure our microgel in the collapsed state, the results obtained here present too high uncertainty to be used to discuss possible effects of the dispersion medium.

Next, we investigated whether electrochemical conditions further modulate the collapsed-state size and architecture of the microgels. To this end, we fixed the temperature at 43 °C (above the VPTT) and varied the dispersing medium between buffer at pH 4, Milli-Q water and buffer at pH 7.

**Figure 7:**
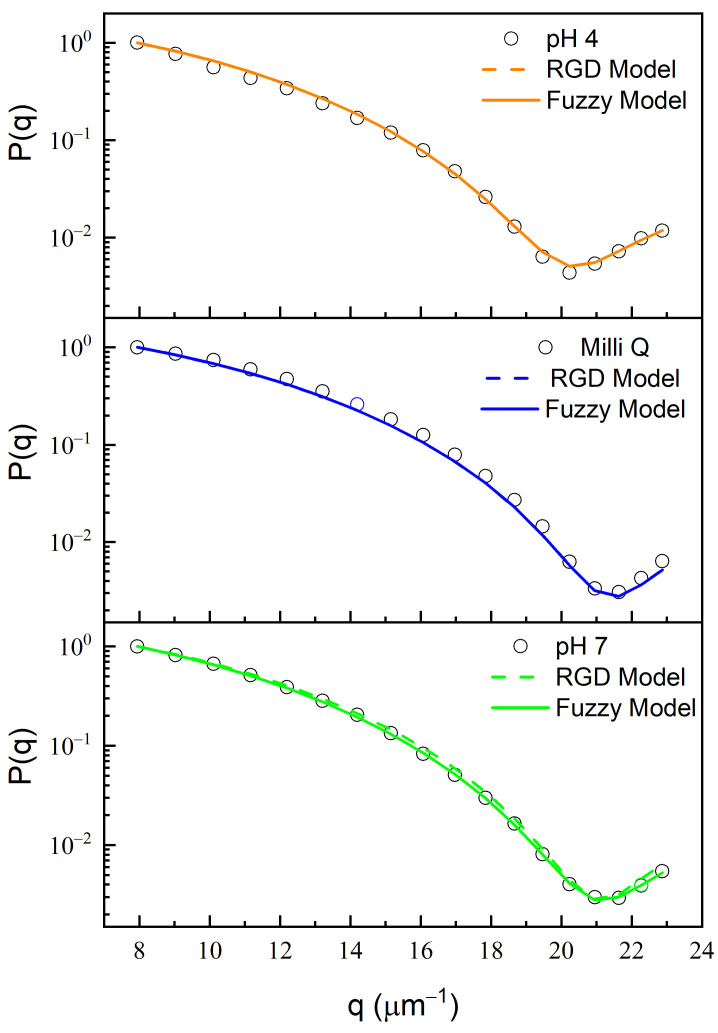
Form factors of microgels in the collapsed state (43 °C) in different media: pH 4 (**top panel**), Milli-Q water (**middle panel**) and pH 7 (**bottom panel**). The solid lines correspond to the RGD model and the dash lines to the fuzzy-sphere model.

Figure 7 shows the static form factors measured at 43 °C in these three media together with fits to the RGD and fuzzy-sphere models. At pH 4 and in Milli-Q water, the two models yield essentially identical radii (R_RGD_ ≈ R) and the shell-width parameter is σ_fuzzy_ ≈ 0 nm, indicating that both core and corona have collapsed into a nearly homogeneous particle. At pH 7, the fuzzy fit yields a slightly different radius and a finite but very small shell thickness σ_fuzzy_ ≈ 1 nm, corresponding to an effective static radius R_SLS_ = R + 2σ_fuzzy_ = 214.0 ± 0.7 nm (Table 3). This residual σ_fuzzy_, on the order of only a few nanometers, still points to a highly collapsed, densely cross-linked network in all cases, with only a marginally more diffuse periphery at pH 7.

Although σ_fuzzy_ ≈ 1 nm is virtually negligible from the point of view of the static form factor, the hydrodynamic radius remains markedly larger than the static radius. This finding is consistent with the presence of a very thin layer of dangling corona chains that remains hydrodynamically active [39,45,46]. Stieger et al. [40] showed that, for PNIPAM microgels cross-linked with BIS ≥ 5 mol %, the polymer density decays approximately linearly within the core and then exhibits an exponential fall-off in the corona, giving rise to a pronounced fuzzy shell in the swollen state that vanishes upon collapse. In contrast, Fernández-Nieves et al. reported a residual shell thickness of σ ≈ 20 nm for microgels prepared with only 2.5 mol % BIS [39]. Within this context, our fitted σ_fuzzy_ ≈ 1 nm supports the picture of a highly collapsed, densely connected network at 43 °C, reflecting either an effectively high cross-link density or a near-complete evacuation of dangling chains from the region that contributes significantly to the static scattering signal. Overall, SLS shows that the core dimensions remain largely unchanged upon varying pH at 43 °C, while any subtle pH-induced restructuring is confined to the outermost shell.

**Figure 8:**
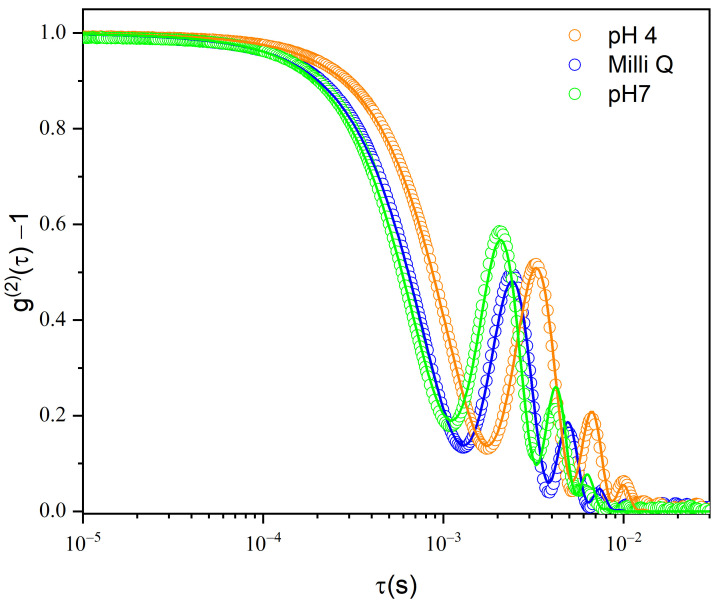
Intensity correlation function of microgels at 43 °C in different media: pH 4, Milli-Q water and pH 7. The solid line corresponds to the convection-corrected model.

Figure 8 presents the intensity autocorrelation functions measured at 43 °C for pH 4, Milli-Q and pH 7. In all three media the correlation function exhibits the two-regime behavior: initial exponential decay at short delay times (Brownian diffusion) followed by pronounced oscillations at longer times (convective drift). Fits using the cumulants approach (R_h_^cum^) and the convection-corrected model (R_h_^conv^) yield the radii listed in Table 3. Although R_h_^cum^ and R_h_^conv^ agree within the experimental error for each condition, only the convection-corrected model captures the oscillatory tail, confirming the drift correction is required for a faithful description of the dynamics. Compared to pure water, where R_h_^conv^ = 265 nm, the convection-corrected hydrodynamic radius increases to 367 nm at pH 4 and 362 nm at pH 7, i.e., by about 100 nm in both cases. Thus, even in the collapsed state, electrostatic interactions and pH-dependence solvation drive an additional swelling of the hydrodynamic microgel size beyond purely thermal collapse. The similarity of R_h_^conv^ at pH 4 and 7 is consistent with that found in the mobility values (Table A2).

The characteristic oscillation frequency, ω = Δq·v, reveals convective velocities of 1.3 mm/s (pH 4), 1.8 mm/s (Milli-Q), and 2.0 mm/s (pH 7). Small shifts in oscillation period reflect slight differences in the vertical temperature gradient across the measurement cell. Although the scattering volume at the bottom of the cell is held at a constant 43 °C, the cell’s upper region remains at ambient temperature, so differences in room temperature produce distinct thermal profiles in each experiment [47]. Therefore, reliable DLS measurements demand control of temperature not only at the dispersion site but across the entire measurement cell.

To compare static and dynamic dimensions across different pH values, we again used the effective static radius from SLS, RSLS, and the convection-corrected hydrodynamic radius R_h_^conv^. The resulting static-to-hydrodynamic size ratios at 43 °C were R_SLS_/R_h_^conv^ ≈ 0.79 (Milli-Q), R_SLS_/R_h_^conv^ ≈ 0.60 (pH 4) and R_SLS_/R_h_^conv^ ≈ 0.59 (pH 7). The decrease from ≈0.79 in Milli-Q water to ≈0.60 at pH 4 and pH 7 indicates that, while the dense core detected by SLS is only weakly affected by pH, the hydrodynamic boundary moves outward due to pH-induced swelling of the outer network. At pH 7, deprotonation of copolymerized carboxyl groups generates internal electrostatic repulsion that drives network expansion; at pH 4, most acid groups are protonated, but enhanced hydrogen bonding between the solvent and polymer increases network hydration [48]. In both cases the electrochemical environment introduces an additional swelling mechanism superimposed on the thermal collapse, thereby fine-tuning the collapsed-state architecture of the PNIPAM microgels.

The fact that R_h_^conv^ remains larger than R_SLS_ for all pH values is consistent with the physical picture discussed above for Milli-Q water (Section 2.1.2): DLS senses an extended hydrodynamic boundary that includes the dilute outer corona and the dragged solvent, whereas SLS is mainly sensitive to the dense-core region.

For comparison with previous work, we interpreted our R_SLS_/R_h_^conv^ values as effective analogs of R_g_/R_h_ and compared them with the typical R_g_/R_h_ range reported for soft colloids [39,41,42]. In our case, the effective static-to hydrodynamic ratios of R_SLS_/R_h_^conv^ ≈ 0.59–0.79 at 43 °C are fully compatible with the literature values of R_g_/R_h_ ≈ 0.60–0.80 for slightly cross-linked PNIPAM microgels and down to ≈0.60 for protein nanogels and collapsed PNIPAM microgels with lower cross-linker content. Moreover, the ratios reported in this study follow the anticipated trend: a larger ratio for thermally collapsed microgels in pure water, where the particles behave as more compact structures, and a smaller ratio at pH 4 and pH 7, where pH-induced charging and enhanced hydration re-establish a softer outer shell. This evolution agrees with the dense-core/soft-shell scenario deduced from SLS, DLS and AFM, and underscores the role of the electrochemical environment (through charge regulation and specific solvent-polymer interactions) in subtly reshaping the architecture of PNIPAM microgels in the collapsed state.

Finally, when comparing NTA and 3D DLS results, a clear discrepancy emerges; however, upon closer examination of the underlying principles of each technique, the findings are entirely consistent. In the collapsed state (T ≈ 43 °C), the microgels follow the systematic trend: R_h_^conv^ > R_NTA_ > R_SLS_. DLS is intensity weighted (∝R^6^), thus overemphasizing larger particles and capturing both the residual solvation layer and any dangling polymer yielding the highest hydrodynamic radius [49]. NTA tracks individual particles to give a number weighted size distribution that reduces the large particle bias of DLS, resulting in an intermediate radius [47]. Finally, SLS fitted with the fuzzy-sphere model reports essentially the high density “core” of the collapsed gel (σ_fuzzy_ ≈ 0), virtually excluding solvation effects and thus yielding the smallest geometric radius [45]. Taken together, these complementary techniques delineate a consistent picture of a compact core surrounded by a pH-sensitive, hydrodynamically active shell.

In brief, when the pH of the medium affected the electrokinetic behavior of the microgel, assessing the effect of the medium on colloidal stability proved relevant in the present system. In this context, 3D-DLS combined with the convection-corrected model provided the most informative hydrodynamic size analysis, while combining these results with the static radius obtained from SLS is highly effective for elucidating the microgel structure.

### 2.2. Drug Loading and Encapsulation Efficiency

To optimize the protocol for drug loading in microgels attending the different strategies reported in the literature, we analyzed different aspects related to it, using the commercial DOX (com-DOX) as the drug. We set the amount of microgel to 0.294 mg.

We repeated the steps of the loading experiment using only microgel, without the presence of drug, and found that two centrifugation cycles of 12,000 rpm, 40 min, at 4 °C were necessary to eliminate residual absorbance. Thus, we fixed these conditions for the centrifugation step.

Filtering of the supernatant proved problematic under our conditions and was therefore avoided, as we found that about 75% of com-DOX was retained in the filter (the pore size was sufficiently small, 0.1 µm, to ensure that only the drug would pass through). This membrane effect could also be present when the drug loading is determined by dialysis [8].

On the other hand, we quantified the effect of performing the loading in the dark or in the presence of light. In this last case, there was a decrease in the absorbance of the supernatant by the effect of the light over the drug, which led to an erroneous determination of the concentration of encapsulated drug (double of that obtained in the dark). Therefore, both encapsulation and release experiments were carried out in darkness, as this proved necessary under our conditions.

We compared the encapsulation experiments at T_encap_ = 25 °C using microgel drug mass ratios of 2:1, and 1:1. Although the encapsulation efficiency (EE) was double in 2:1 case, the drug loading (DL) was similar in both cases (DL = 6%). Thus, we chose the 1:1 condition, as less microgel was used to load the same amount of drug per particle.

Regarding the temperature of encapsulation, we compare the results obtained at T_encap_ = 25 °C and 32 °C (near the VPTT of PNIPAM) using a microgel:drug mass ratio of 1:1 (EE and DL agree in this case). At T_encap_ 32 °C, the DL was 23%. This is higher than that obtained at room temperature (DL = 6%). This can be explained by analyzing drug–microgel interactions. The electrostatic attractive force between the positive charges of DOX and the negative carboxylic groups of the PNIPAM-co-COOH microgel is expected to play an important role in promoting the diffusion and interaction of drug molecules with the microgel network [50,51].

The electrokinetic charge of the microgel at 25 and 32 °C is similar at pH 7, as shown in Figure 6. Therefore, the DOX–microgel electrostatic attraction should be similar at both temperatures. However, although DOX is considered a hydrophilic drug, it also exhibits a hydrophobic character that is increased at a neutral pH [52] and, since the loading was performed in a neutral pH medium, the DOX–microgel hydrophobic interaction will also play a role [53]. At T_encap_ = 32 °C, the interior of the microgel is more hydrophobic, as it is near the VPTT, then, that hydrophobic drug–microgel attraction will be higher than at T_encap_ = 25 °C.

Another aim in our drug loading studies was to compare UV-Vis and HPLC techniques for drug concentration measurement. The drug concentration in the supernatant of the loading experiments resulted well above the maximum detection limit in HPLC. Thus, UV-Vis spectrophotometry proved to be a more suitable technique under the concentration range of the present loading experiments.

Once the optimal encapsulation protocol was established (T_encap_ = 32 °C, darkness, mass ratio 1:1) the procedure was scaled up by setting the amount of microgel to 3 mg, and encapsulated the drugs 5FU and DOX. The final drug loading (DL = EE in this case) was 14% for 5FU and 65% for DOX. This difference was due to the electrostatic attraction of the positive DOX and the negative PNIPAM, and the higher hydrophobicity of DOX at neutral pH, as the microgel near the VPTT results in more hydrophobic, as previously commented. Additionally, the H-bonds between the protonated amine groups and hydroxyl hydrogens of DOX and H-bond acceptors of the polymeric chains (such as the carbonyl groups) might also be an important force for the successful encapsulation of DOX inside the microgel particles [54]. On the other hand, 5FU molecules result attached to PNIPAM surface by means of both, hydrogen bonding and hydrophobic interactions [55,56,57,58].

It should be highlighted that the real encapsulated drug percentage is higher in all cases, as the centrifugation of the sample (necessary for the determination of the free drug in the supernatant) forces the release of part of the encapsulated drug (as detected later in the release experiments) [30].

Finally, we verified that after two months of refrigerated storage (4 °C), practically all of the drug remained encapsulated in the nanoparticles. Only 14% of the encapsulated drug was released (even less due to the centrifugation effect in its determination).

In brief, we used the standard method of incubation of the drug/microgel mixture at the encapsulation temperature (T_encap_) followed by centrifugation. Next, the concentration of drug in the supernatant was measured. The procedure to correctly determine the amount of loaded drug and the drug encapsulation conditions to get the highest drug loading have been optimized: (i) to eliminate residual absorbance in the supernatant coming from the microgel, the centrifugation conditions should be fixed using a microgel blank; (ii) filtering of the supernatant should be avoided; (iii) both loading and release should be performed in darkness; (iv) UV-Vis spectrophotometry is a better choice to determine the drug concentration in the supernatant of the encapsulation experiments than HPLC; (v) the mass ratio 1:1 is recommended as less microgel is used to encapsulate the same amount of drug per particle; (vi) the hydrophobic interaction between the drug and the microgel plays an important role and determines an optimal encapsulation temperature close to the VPTT of the microgel (T_encap_ = 32 °C); (vii) the incubation–centrifugation procedure may lead to an underestimation of the amount of encapsulated drug due to forced diffusion during the centrifugation step; and (viii) the existence of attractive interaction between the drug and the microgel makes it possible for the DL to decrease only slightly after one month of storage.

### 2.3. Drug Release

Initially, we carried out the release experiments using the incubation/centrifugation method. In this experiment we used the commercial DOX. The percentage of the drug released over the drug encapsulated as a function of time was the same after 15 min than after 1440 min, which could be explained by the forced release of the drug when the sample is centrifuged. Therefore, this method is not appropriate to model the kinetics of release of drugs in microgels. However, it is useful to obtain experimental information on the drug/microgel interaction as explained below. When com-DOX encapsulation was performed at 32 °C, the released/encapsulated percentage of drug was 5%, which is lower than the 18% found for com-DOX encapsulated at room temperature. This shows a significantly stronger drug–microgel interaction in the former case, which reinforces the important role that hydrophobic interaction plays between DOX and the less swollen microgel, as found with DL results. However, as the DL was higher at 32 °C (DL = 23%), the net amount released of DOX was still slightly higher in this case.

Regarding the dialysis release method, we firstly checked that the absorbances of the solutions of DOX and 5FU did not change along time at the encapsulation conditions (buffer pH7, T = 37 °C). Thus, these drugs were stable under such conditions. Then, we tested whether the membrane could generate a delay in drug diffusion. For this purpose, we analyzed the diffusion of both free drugs deposited inside different dialysis membranes (up to 300 kDa). We measured the amount of drug in the outer volume (by UV-Vis spectrophometry) and found that the membrane produced a delay in diffusion that could affect the drug release analysis at short times [25,,,59].

**Figure 9:**
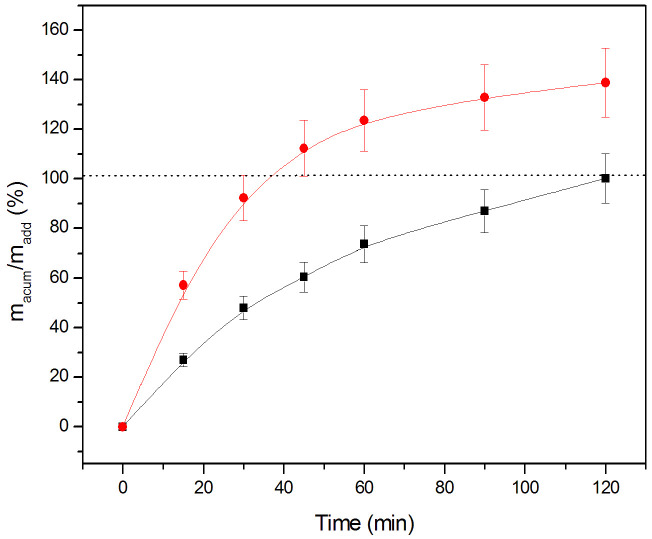
Comparing absorbance and HPLC: Percentage of accumulated 5FU in the external volume of the dialysis experiment over the free 5FU added in the internal volume as a function of time. Method of determination of drug concentration: absorbance (circles), HPLC (squares).

To quantify the effect of drug delay due to the dialysis membrane during the release experiment, we performed the dialysis protocol described for drug-loaded microgels in Section 4.5.2, but added 2.5 mL of free drug inside the dialysis tube in the absence of microgel particles. We removed all the external volume, as we will later show that it led to less experimental artifacts. In addition, we used these experiments to compare the results of the drug accumulated in the external volume analyzed by UV-Vis spectrophotometry (absorbance) and HPLC (the results for 5FU are shown in Figure 9). In this figure, we can observe discrepancies between the two analyses from the very first minutes. The value obtained by absorbance was always higher than by HPLC and resulted in failed cumulative mass percentages higher than 100%. The reason is that the real drug concentration was so low that the absorbance technique was not sensitive enough. Thus, we decided to establish HPLC as the technique of determination of the concentration of the drug in the release dialysis method.

**Figure 10:**
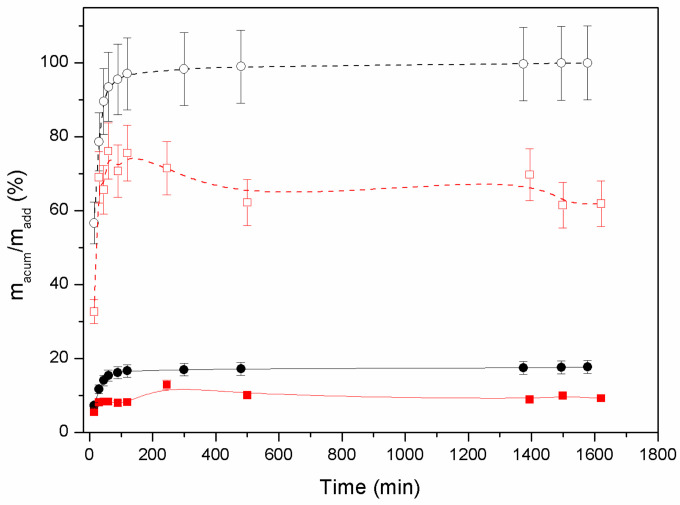
Percentage of accumulated DOX in the external volume of the dialysis experiment over the DOX added (free DOX, open symbols) or encapsulated (microgel-DOX complex, solid symbols) in the internal volume as a function of time. Retiring/replacing all external volume (circles) or only a part (squares).

We also analyzed the effect of removing all or only 0.5 mL of the external volume and replacing the same volume of fresh buffer solution. As shown in Figure 10 for DOX, unexpected irregular behaviors for both the dialysis of the free drug and the drug–microgel complex were found. A similar behavior for 5FU was obtained. In dialysis experiments, sink conditions in the external container are normally maintained to make back diffusion negligible. This sink condition is reached when the drug concentration in such a container does not exceed 10% of that in the internal container [25]. According to our experimental results, this sink condition was fulfilled when the entire volume of the external container was removed. This can be verified by the free DOX curve, which has the highest value of mass released at the first collection point. However, when only a part of the volume in the external compartment was removed, we found artifacts in the curves, which may be due to back diffusion. We conclude that removing only part of the external volume generates more experimental artifacts.

**Figure 11:**
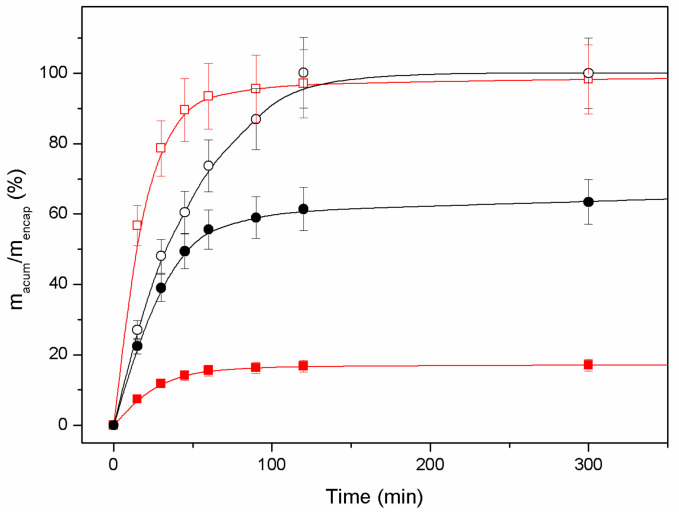
Percentage of accumulated drug in the external volume of the dialysis experiment over the encapsulated drug in the microgel added in the internal volume as a function of time. Free drugs (open symbols) and microgel-released drugs (solid symbols). DOX (squares); 5FU (circles).

By establishing HPLC as the drug analysis method and the dialysis process by retiring/replacing all the external volume, we present the results of release of 5FU and DOX from the microgel. In Figure 11 we compare the percentage of drug (5FU (circles) or DOX (squares)) accumulated in the external volume over the drug added (free drug) or encapsulated (microgel–drug complex) in the internal tube.

Firstly, we quantified the delay of the free drugs due to the dialysis membrane. 5FU is neutral and smaller in size than DOX, so it would be expected to diffuse more rapidly across the membrane. However, as the amount of drug inside the dialysis tube was much lower (DL = 14% for 5FU versus 65% for DOX), the concentration gradient across the membrane is smaller for 5FU, so the flux will also be smaller according to Fick’s law.

Since the dialysis method introduces a delay in the release of the drug, the times shown on the X-axis are not the actual drug release times from the microgel. Therefore, for precise drug release times, corrections should be made to account for the time delay experienced by the drug as it passes into the external volume.

It is noteworthy that for 5FU, the first point of the curve is similar in the cases of the free and encapsulated drugs. This indicates rapid release (burst release effect) of the drug from the nanoparticle in the initial moments due to the shrinkage of the microgel (there exists a change in the temperature of the sample from room conditions to 37 °C) and without the effects of the drug–microgel interaction. Once the particle collapses, the process is influenced by the passive diffusion of the drug into the microgel, which drastically slows because of the reduction in the mesh size and a denser and more compact surface layer of the microgel [21,60,61]. Since the release rate drops sharply as the hydrogel empties, the First-Order Model typically fits curves with early plateaus (initial burst release) very well assuming that the release rate is directly proportional to the remaining 5FU concentration in the hydrogel [61].

On the other hand, the higher attraction of the DOX with the microgel due to electrostatic and hydrophobic contributions explains the differences found between the free and the encapsulated DOX in the first instants of the release curve and the low degree of cumulative release achieved, around 20%, which is typical for this drug if the release conditions are similar to physiological ones [51,62,63].

Zambito et al. [25] used drug-loaded chitosan nanoparticles crosslinked with tripolyphosphate to show that dialysis data from a nanoparticle dispersion is not necessarily descriptive of sustained release from nanoparticles. They used a MWCO 12 kDa membrane and drugs of around 300 Da. The process was in fact controlled by permeation across dialysis membrane. Some authors use models to correct the dialysis-derived drug release curve from other types of nanoparticles by using the dialysis data of the free drug. However, this requires measurement of the drug concentration in the donor compartment [25,27].

To sum up, the incubation/centrifugation method is unsuitable to model the kinetics of release of drugs in these microgels. However, it serves to obtain comparative experimental information on the drug/microgel interaction. Dialysis provided a more suitable framework in the present system, but considering the following recommendations: (i) it proved necessary to check drug stability under release conditions; (ii) HPLC for the analysis of the drug concentration and the replacement of all external volume in the outer compartment are found to be the most suitable methods to determine drug concentration in dialysis release experiments; and (iii) dialysis membrane causes drug delay: a free-drug blank is necessary to obtain correct data on early drug release kinetics in these microgels.

## 3. Conclusions

This work systematically examines several protocol-dependent effects encountered during the colloidal characterization of thermosensitive PNIPAM microgels, as well as during the evaluation of their drug loading and release behaviors.

Although the experimental protocol-dependent effects discussed here are broadly relevant to thermosensitive microgels and other soft nanocarriers, the magnitude of the observed biases should not be considered universal. It is expected to depend on both particle-specific and protocol-specific factors. Relevant particle-related parameters include particle size, optical contrast, polydispersity, softness, permeability and the degree of swelling, all of which may change with temperature, pH and ionic strength. In light scattering measurements, the magnitude of the bias also depends on sample volume, cell geometry, thermal equilibration, the presence of vertical temperature gradients and the optical configuration used to collect the correlation function. In loading and release experiments, additional factors such as microgel concentration, drug identity and concentration, drug–polymer affinity, centrifugation conditions, membrane chemistry, membrane area, inner/outer volume ratio and sampling/replacement protocol may affect the measured values. Therefore, the recommendations derived from this work should be applied to other systems only after explicitly reporting these parameters and, when necessary, re-optimizing the corresponding experimental protocol.

In this PNIPAM-co-COOH microgel system, combining complementary techniques was essential to avoid misleading interpretations based on a single standard method.

Image techniques such as TEM and AFM were useful for assessing particle shape and polydispersity, whereas SLS provided the most informative quantitative description of the static core–corona structure in dispersion. Because these techniques probe different physical observables, their size values should not be directly equated.

For hydrodynamic sizing, NTA provided size distribution only in the collapsed state, where the optical contrast was sufficient for tracking. The standard single-beam DLS configuration used here did not yield reliable size estimates for this microgel. At 43 °C, residual convective drift associated with a vertical temperature gradient across the cell caused a purely diffusive cumulant analysis to underestimate the collapsed hydrodynamic radius by up to 18%. The drift-corrected 3D-DLS analysis, combined with SLS, provided a consistent description of the thermally induced collapse, the pH-dependent swelling and the hydrodynamic boundary of the particles.

Concerning drug loading, the standard method of incubation of the drug/microgel mixture followed by centrifugation has been revised and the procedure to correctly determine the amount of loaded drug has been optimized. Centrifugation conditions should be fixed using a microgel blank to remove residual absorbance; supernatant filtering should be avoided; and loading/release experiments should be performed in darkness. In our study, the encapsulation conditions have also been optimized to achieve the highest drug loading (DL) values for these hydrophilic drugs and the PNIPAM-co-COOH microgel used. The hydrophobic drug–microgel interaction plays an important role and determines an optimal encapsulation temperature close to the VPTT of the microgel (T_encap_ = 32 °C). The incubation–centrifugation procedure may lead to an underestimation of the amount of encapsulated drug due to forced diffusion during the centrifugation step and this limitation should be considered when comparing drug loading values across studies.

For drug release studies, the incubation/centrifugation method was determined to be unsuitable for modeling release kinetics in these microgels although it still provided comparative information on drug–microgel interactions. In contrast, dialysis offered a more appropriate framework but several aspects must be considered: (i) drug stability under release conditions should be verified; (ii) sufficiently sensitive analytical methods (HPLC in our case) and the replacement of all external volume are found to be the most suitable methods to determine drug concentration in dialysis release experiments; and (iii) dialysis membrane can introduce a drug delay, so a free-drug blank under identical conditions is essential for interpreting the early-time release kinetics.

Overall, this study shows how protocol-dependent effects can bias characterization, loading and release measurements in a PNIPAM-co-COOH microgel system and provides an experimentally grounded framework for interpreting similar measurements in related thermosensitive microgels. The extent to which these observations can be transferred to other systems will depend on particle size, optical contrast, polydispersity, medium composition and temperature-control geometry.

## 4. Materials and Methods

### 4.1. Materials

N-Isopropylacrylamide (NIPAM), 3-butenoic acid (3BA), methylenebisacrylamide (BIS), 2,2′-azobis(2-methylpropionamidene) (V50), and isopropanol (99.8%) were supplied by Aldrich. Firstly, for the optimization of the protocol of the drug encapsulation, we used commercial Doxorubicin hydrochloride (Doxorubicina Accord 2 mg/mL, com-DOX). Once the protocol was established, we chose the drugs 5-fluorouacil (5FU) and Doxorubicin hydrochloride 98.0–102.0% (HPLC) (DOX) supplied by Merck Life Science S.L. (Madrid, Spain). All reagents were used without further purification. Water was purified in a Milli-Q Academic Millipore system. When necessary, sample pH was controlled using a buffer solution (borate (5.14 mM H_3_BO_3_) for pH 9, phosphate (1.13 mM NaH_2_PO_4_) for pH 7, and acetate (3.15 mM AcH) for pH 4).

### 4.2. Formulation of the Microgels

Following the protocol published by García-Pinel et al. [7], copolymeric nanoparticles (microgels) were prepared by mixing the monomer NIPAM (3 mmol, 0.339 g) and the cross-linker BIS (0.3 mmol, 0.046 g) in Milli-Q water (20 mL). When the reaction temperature was reached (70 °C), the co-monomer 3BA (0.35 mmol, 0.03 g, 0.03 mL) and the radical initiator V50 (100 mM, 0.150 mL) were sequentially added. For cleaning, the sample was subjected to several cycles of centrifugation and re-dispersion in Milli-Q water (4 times at 8000 rpm for 1 h). The last pellet was redispersed in 20 mL of Milli-Q water to produce a clear colloidal dispersion. An aliquot was taken and lyophilized for analysis. The sample was protected from light and stored at 5 °C.

### 4.3. Characterization of the Microgels

#### 4.3.1. Particle Morphology

##### Transmission Electron Microscopy (TEM)

Microgels were imaged by TEM using the microscope Libra 120Plus (EDX, 120 kV, Carl Zeiss SMT, Oberkochen, Germany) from the Centre for Scientific Instrumentation of the University of Granada (CIC, UGR). Sample preparation was also performed at the CIC, UGR: 30 microliters of the suspension (microgel redispersed in buffer pH 7) was incubated on a grid with charcoal support film for 5 min in a Petri dish. The grids were dried on filter paper and subsequently oven dried at 37 °C for 2 min. A size histogram was obtained by measuring over 100 individual particles (automatically analyzed with Bolero software, Bool2k v. 02, AQ Systems).

##### Langmuir–Blodgett Deposition and Atomic Force Microscopy (AFM)

To deposit microgels from Milli-Q water/air interfaces into silicon substrates, we used a Langmuir trough (KSV NIMA, Biolin Scientific, Gothenburg, Sweden) made of Teflon, equipped with two motorized barriers made of Delrin, and a dipping arm. Prior to each experiment, the trough and barriers were thoroughly cleaned in a sequence involving rinsing with tap water, distilled water, wiping with Kimtech paper and isopropanol, distilled water, and Milli-Q water. Silicon substrates (<100> p-type boron doped, 1–10 Ω·cm, University Wafer Inc, South Boston MA, USA.), cut into 2 × 1 cm^2^ pieces using a laser cutter (E-20 SHG II, Rofin, Hamburg, Germany), were used for the deposition.

The first step involved lowering the substrate into the trough with the dipping arm, positioned at a 60° angle relative to the interface. The trough was filled with Milli-Q water, with the barriers in the open position. After 10 min the paper Wilhelmy plate was fully wetted, and the surface pressure (Π) was set to zero. The interface was then cleaned by closing the barriers and removing the impurities at the interface using a vacuum pump and a clean pipette tip. This process of barrier closure, surface cleaning, and resetting Π to zero was repeated until Π ≤ 0.2 mN/m after full compression, leaving the barriers open.

Next, isopropanol was added to the microgel dispersion as a spreading agent, up to a 10:1 water:isopropanol ratio. A total of 500 µL of the microgel dispersion was then deposited at the interface using a glass microsyringe, and the system was allowed to equilibrate for 10 min. To prevent contamination from dust and air currents, the trough was covered with a transparent case. The monolayer was then compressed to 5 mN/m at 5.2 mm/min, after which the substrate was raised using the dipper arm at a speed of 0.5 mm/min.

The resulting monolayers were characterized by atomic force microscopy (AFM, Dimension 3000, Bruker (Veeco/DI), Billerica, MA, USA) in tapping mode using Tap300Al-G cantilevers (300 kHz, 40 N/m, BudgetSensors, Sofia, Bulgaria). AFM images were acquired at a resolution of 512 × 512 pixels^2^ over a 40 × 40 µm^2^ area. The images were leveled using Gwyddion software (version 2.70) to ensure accurate analysis. This approach allowed us to obtain profiles of individual microgels. Gaussian fits were then applied to determine the diameter and standard deviation of the deposited microgels, providing evidence of their core–shell structure. These experiments were conducted at room temperature.

##### Static Light Scattering (SLS)

To obtain information on the morphology of the microgel we used a 3D light scattering instrument (LS Instruments AG, Fribourg, Switzerland) which is widely employed to characterize both dilute and concentrated colloidal suspensions. Under the low-concentration conditions used here, interparticle contributions are expected to be small. Therefore, the analysis is interpreted mainly in terms of the single-particle form factor, P(q). This assumption should be understood as an approximation valid for the present concentration range and experimental conditions.

**Figure 12:**
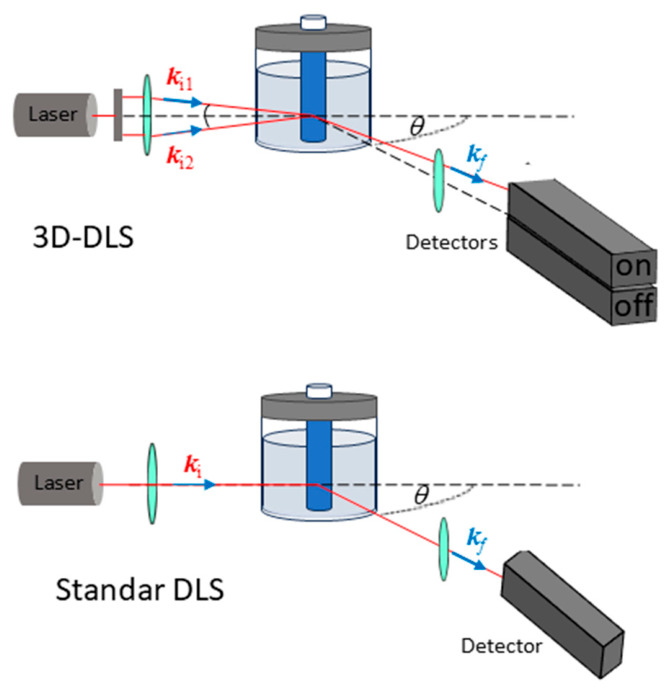
Scheme of the setup for 3D-DLS (**up**) and the standard DLS (**down**).

The setup is equipped with a He-Ne laser (wavelength λ = 633 nm, LS Instruments AG, Fribourg, Switzerland). The primary laser beam is split into two beams that intersect in the sample and are displaced symmetrically above and below the scattering plane (see Figure 12). In the configuration used here, only the upper detector is active and collects the scattered intensity arising from its corresponding incident beam, while it also receives a small contribution from light scattered from the second beam. The sample was contained in a cylindrical optical glass cell with an internal diameter of 10 mm, positioned at the center of a vat filled with cis/trans decalin to minimize stray-light contributions. The temperature was controlled with an external thermostatic bath and a calibrated temperature sensor. Additional details of the 3D-DLS setup and its optical geometry can be found in López-Molina et al. [38].

Dilute suspensions of PNIPAM microgel were prepared at room temperature. Each sample was measured at least three times independently under controlled temperature conditions, and the resulting intensity curves were averaged to improve the signal-to noise ratio and ensure reproducibility. The measurements were performed over a range of scattering angles (θ) from 20° to 140°, which provides access to the angular dependence of the scattered intensity, I(q). For dilute suspensions, when I(q) is normalized by the incident intensity I(0), the experimental form factor is obtained as P(q) = I(q)/I(0) [47].

The static radius is then extracted by fitting the experimental form factor to different theoretical models. Within the Rayleigh–Gans–Debye (RGD) approximation, the form factor of a homogeneous sphere of radius R is given by

(1) $$P\left(q\right)={\left(\left(\frac{3}{{\left(qR\right)}^{3}}\right)\left(sin\left(qR\right)-qRcos\left(qR\right)\right)\right)}^{2}$$

where q is the scattering vector, defined as q = (4π/λ) sin(θ/2), with n being the refractive index of the solvent and θ being the scattering angle. The RGD model assumes a uniform distribution of scattering material within the sphere. However, thermosensitive microgels typically exhibit an inhomogeneous cross-linking density, with a denser core and a more diffuse corona.

**Figure 13:**
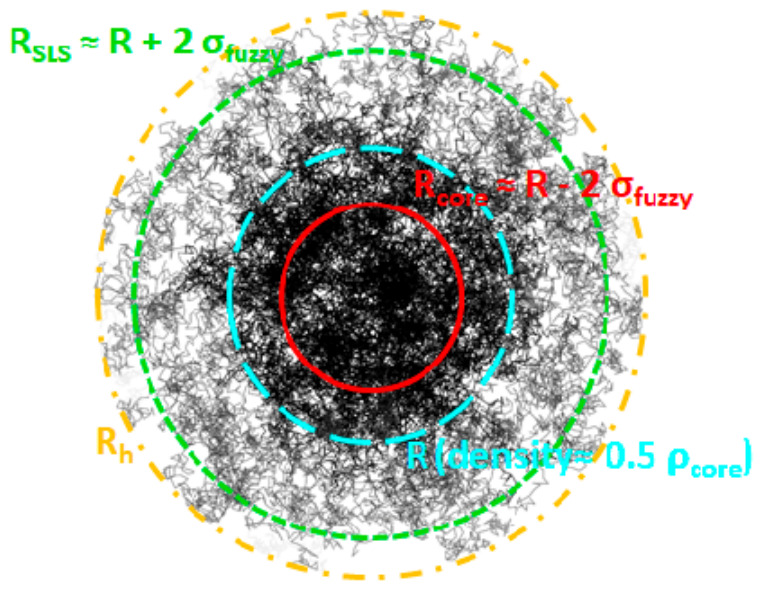
Schematic PNIPAM microgel with core radius R_core_ ≈ R − 2σ_fuzzy_, intermediate radius R, effective static radius R_SLS_ ≈ R + 2σ_fuzzy_ and hydrodynamic radius R_h_, illustrating that R_h_ extends beyond the static boundary.

To account for this radial inhomogeneity, Stieger et al. [40] introduces the so-called fuzzy-sphere model, in which the homogeneous sphere of radius R is convoluted with a Gaussian of width σ_fuzzy_. The inner core with nearly constant polymer density extends up to R_box_ ≈ R − 2σ_fuzzy_, the density has decayed to about half of the core value at r = R, and it approaches zero around R_SLS_ ≈ R + 2σ_fuzzy_, which can be taken as the overall particle radius obtained from SLS (Figure 13). This leads to a form factor, as shown in the following equation:

(2) $$P\left(q\right)={\left(\left(\frac{3}{{\left(qR\right)}^{3}}\right)\left(sin\left(qR\right)-qRcos\left(qR\right)\right)\times exp\left(-\frac{{\left(q\sigma_{fuzzy}\right)}^{2}}{2}\right)\right)}^{2}$$

In this model the fitting parameters are the radius R and the smearing parameter σfuzzy, which is approximately half the thickness of the fuzzy shell. The simplicity of this analytical expression largely explains the success of the fuzzy-sphere model in describing microgels, even though it is known that it does not strictly enforce a decay of the density to zero at a finite distance from the particle center.

#### 4.3.2. Hydrodynamic Size

Complementary techniques (NTA and DLS) were used to determine the hydrodynamic size of the microgel system. These techniques analyze the Brownian motion of the particles and convert the diffusion coefficient (D) into particle size using the Stokes–Einstein equation [47].

(3) $$D=\frac{k_{B}T}{6\pi \eta R_{h}}$$

where R_h_ is the hydrodynamic radius, k_B_ is the Boltzmann constant, T is the temperature and η is the solvent viscosity.

In preliminary measurements, we verify that it was necessary to sonicate the sample to disperse the particles correctly before measuring. To perform valid comparative studies, we set the following conditions: a microgel concentration of 5.88 10–3 mg/mL in Milli-Q water, and a controlled sonication time. In the Section 2, we showed (via NTA experiments) that the optimum sonication time for our microgel was 2 h and fixed it for all measurements of the hydrodynamic size. Once the sample was introduced in the device, we waited 10 min before measuring so that the system could adjust to the temperature.

##### Nanoparticle Tracking Analysis (NTA)

The hydrodynamic size distribution of the microgel was measured by using nanoparticle tracking analysis (NTA) in a NanoSight LM10-HS(GB) FT14 (NanoSight, Amesbury, United Kingdom) [47]. All samples were measured more than three times for 60 s with manual shutter, gain, brightness, and threshold adjustments at a fixed temperature. The average size distribution (particle concentration vs. diameter) was calculated as an average of at least three independent size distributions. When comparing results, it is necessary to use the same capture parameters (Camera Level, CL) and analysis parameters (Detection Threshold, DT).

##### Standard DLS Characterization

The hydrodynamic size was also characterized by dynamic light scattering (DLS) using a Zetasizer NanoZeta ZS device (Malvern Instrument Ltd., Worcestershire, U.K.) with a He-Ne laser of 633 nm and a scattering angle of 173°. The average hydrodynamic diameter (Z-average or cumulant mean) and the polydispersity index (PDI) were computed. These parameters are calculated through a cumulant analysis of the data, which is applicable for narrow monomodal size distributions [64]. The intensity size distribution can also be determined from an algorithm provided by the Zetasizer software (version 7.13, General Purpose). In addition, we used a Zetasizer Ultra device (Malvern Instrument Ltd., U.K.) with a capillary cell which is indicated for measuring big nanoparticles.

##### 3D-DLS Characterization

The hydrodynamic radius of the microgel particles was determined using the 3D-DLS setup described in Section Static Light Scattering (SLS). The time-averaged intensity autocorrelation function, g_2_(τ) − 1, was measured at a scattering angle of θ = 30°. This quantity is linked to the electric-field correlation function, g_1_(τ), via the Siegert relation (g_2_(τ) – 1 = |g_1_(τ)|^2^), which holds when the scattered-intensity fluctuations are Gaussian [47,49].

In dilute conditions, interparticle interactions can be neglected, so the field autocorrelation function g_1_(τ) reflects only the Brownian motion of individual microgels. Under these circumstances, the Siegert relation leads to g_2_(τ) − 1≈ exp(−2Dq^2^τ), allowing the diffusion coefficient D to be extracted from the decay rate. The corresponding hydrodynamic radius, Rh, is then obtained via the Stokes–Einstein equation (Equation (3)).

To account for the polydispersity or peak broadening effects, the cumulants method expands the logarithm of g^1^(τ) as a power series in the delay time, τ.

(4) $$\ln\left|g^{\left(1\right)}\left(q,\tau \right)\right|=-\mu_{1}\tau +\frac{1}{2!}\mu_{2}\tau^{2}-\frac{1}{3!}\mu_{3}\tau^{3}+\ldots$$

where the μ_i_ are the cumulants [65]. In practice, only the first two cumulants (μ_1_ and μ_2_) are reliably determined, and the initial slope, μ_1_, is used to extract D and hence R_h_.

In drug delivery applications, particulate carriers can experience temperature gradients that alter their Brownian motion and thus their apparent hydrodynamic radius. To assess this effect, we examined how a thermal gradient influences DLS measurements of our microgel suspension. Previous work by López-Molina et al. [38] has shown that, under thermal gradients, DLS data become distorted by thermal convection: flow-driven particle drift through the scattering volume induces oscillations in the intensity autocorrelation function, g_2_(τ) − 1, and accelerates its decay, leading to biased size estimates. To remove these artifacts, we applied the modified correlation function introduced by López-Molina et al. [38], which decouples diffusion and drift contributions. In our single-detector setup with two intersecting beams defined by scattering vectors q_1_ and q_2_, the corrected correlation function is:

(5) $$g^{\left(2\right)}\left(\tau \right)-1=e^{-2D\bar{q^{2}}\tau }\left[\left(1-C\right)+C\cos\left(\Delta q\cdot v\tau \right)\right]{\left(1-\frac{v\tau }{h}\right)}^{2}$$

where q =|**q**_1_ + **q**_2_|/2 is the mean scattering-vector magnitude, **Δq** = **q**_1_ − **q**_2_ is their difference, v is the particle drift velocity, h is the beam thickness and C (with 0 ≤ C ≤ 0.50) represents the relative intensity weight of the two beams. The factor (1 − v τ/h)^2^ captures the rate at which particles leave the scattering volume due to convective drift. This correction allowed for a more accurate measurement of the hydrodynamic size by compensating for the artifacts introduced by convection-induced particle motion.

In both models, the hydrodynamic microgel radius was determined by fitting the experimental autocorrelation functions to the respective theoretical models.

#### 4.3.3. Electrokinetic Behavior

The electrophoretic mobility was determined by the technique of Laser Doppler Electrophoresis (LDE) using the Zetasizer NanoZeta ZS device (Malvern Instrument Ltd., U.K.) described above. Each data point was taken as an average over three independent sample measurements. For each measurement, the electrophoretic mobility distribution was considered to get the mean and standard deviation.

#### 4.3.4. Colloidal Stability in Different Media

To analyze the colloidal stability of the microgel nanoparticles, we studied the effect of the dispersion medium characteristics (Milli-Q water or buffer) on the hydrodynamic size, measured by NTA and 3D-DLS, and on the static radius obtained from SLS. We also analyzed the temporal stability of the system by determining the mean hydrodynamic diameter as a function of time with NTA. We set the temperature of the medium to 43 °C, a microgel concentration of 5.88 × 10^−3^ mg/mL, and a sonication time of 2 h before measuring. After introducing the sample into the device, we waited for 10 min to measure so that the system could adjust to the temperature (the reasons for this are discussed in the Section 2). The NTA parameters were fixed to CL = 14 and DT = 5.

### 4.4. Drug Loading and Encapsulation Efficiency

The encapsulation protocol consisted of mixing the aqueous solutions of the microgel and drug (at certain microgel:drug mass ratio) and subsequent incubation for 48 h in darkness at the encapsulation temperature (T_encap_) under continuous stirring (400 rpm) using a thermal shaker (Thermal Shaker Heat/Cool, Parsippany, NJ, USA, Ohaus). The mixture was centrifuged and the concentration of drug in the supernatant was measured using UV-Vis spectrophotometry (Eppendorf, Hamburg, Germany BioSpectrometer) and ultra-high-performance liquid chromatography (UHPLC) in a Waters Xevo Tq-S mass spectrometer (Milford, MA, USA) (Centre for Scientific Instrumentation of the University of Granada). To determine the concentration of drug in the supernatant by UV-Vis spectrophotometry, we measured the spectrum of each drug (com-DOX, DOX and 5FU) to know the wavelength corresponding to the maximum absorbance λ (Abs^max^). Then, we performed the corresponding calibration at λ (Abs^max^).

Firstly, to optimize the procedure of encapsulation of drugs in microgels, we used the commercial DOX and checked the influence of different strategies found in the literature: (i) repetition of the experiment with a microgel blank to establish the centrifugation cycles necessary to remove residual non-drug absorbance; (ii) filtering of supernatant; (iii) performing the incubation in darkness or in presence of light; (iv) different microgel:drug mass ratio (2:1 and 1:1); and (v) different encapsulation temperatures, near the VPTT (T_encap_ = 32 °C) and lower than the VPTT (T_encap_ = 25 °C), due to the thermosensitive property of this microgel.

Once the optimal encapsulation protocol was established, we scaled it and encapsulated the drugs 5FU and DOX to obtain the drug-loaded microgel nanoparticles to be used in the release experiments.

The pellet was finally resuspended in filtered buffer of pH 7. The amount of encapsulated drug was calculated by measuring the difference between the initial mass of added drug (M_I_) and the free non-encapsulated drug (M_F_), this is the total mass of drug in the aqueous supernatant, which was tested by spectrophotometry. Then, drug encapsulation efficiency (EE) and final drug loading (DL) were calculated as follows:

(6) $$EE=\frac{\left(M_{I}-M_{F}\right)}{M_{I}}100$$

(7) $$DL=\frac{\left(M_{I}-M_{F}\right)}{M_{microgel}}100$$

where M_microgel_ is the mass of microgel [33].

Finally, we measured if the drug remained encapsulated after 2 months of storage in a fridge (4 °C) by determining the concentration of drug in the supernatant by UV-Vis spectrophotometry after centrifugation.

### 4.5. Drug Release

Different protocols for the characterization of the release of the drug encapsulated inside microgels were tested in this work.

#### 4.5.1. Incubation/Centrifugation Method

One method was the incubation of the solution with the drug–microgel complex at the desired temperature of release (Trelease) under magnetic stirring. At specific intervals, a certain volume is removed and centrifuged, and the drug concentration of the supernatant is determined. We named this the incubation/centrifugation method [28]. In this experiment we used the commercial DOX. A total of 5 mL of com-DOX–microgel complex solution was incubated at T_release_ = 37 °C. After a certain amount of time (from 15 to 1440 min), 0.5 mL was removed and centrifuged to measure the drug concentration in the supernatant by UV-Vis spectrophotometry. We also compared the release results for microgels loaded at different encapsulation temperatures of 25 °C (room temperature) or 32 °C (near VPTT).

#### 4.5.2. Dialysis Method

Dialysis is a very common method consisting in adding the solution with the drug–microgel complex to a dialysis membrane (with low enough pore size to avoid microgel diffusion), which is inserted into a container with the external buffered solution. The container is kept at 37 °C under shaking. At specific intervals, a certain external volume is removed to measure the corresponding drug released concentration and is replaced by fresh buffer solution [4,7,18,19,21,22,23].

In the dialysis method, several experimental hurdles could influence the results: (i) the passage of the drug through the dialysis membrane can lead to a delay and incorrect interpretation of the experimental results of the drug release time from the microgel. To account for this possible effect, the release process should be repeated, but by adding a buffered solution containing the same amount of drug that was encapsulated inside the microgel inside the dialysis membrane. (ii) The drug could aggregate inside the dialysis membrane. Thus, the temporal stability of the drug solution at the encapsulation conditions (buffer pH7, T = 37 °C) should be analyzed by measuring its absorbance as a function of time. (iii) The relation between the external buffered solution volume and the sample volume inside the dialysis membrane. A higher external volume favors the diffusion process, but the concentration of released drug in such external volume could be too low to be correctly determined. This is especially important when the amount of encapsulated drug is low and when the initial release times are of interest. To reduce the external volume necessary when dialysis membranes are used, low-volume dialysis tubes (Pur-A-Lyzer Maxi 12,000 Da, Sigma-Aldrich, St. Louis, MA, USA) could be an alternative. A volume of 2.5 mL of the drug–microgel complex can be added to a dialysis tube and inserted into a tube containing 15 mL of buffered solution. (iv) The external volume which is removed and replaced. Some authors retire the whole external volume to measure the concentration of released drug, which is replaced by the same volume of freshly buffer solution [33]. However, other authors retire and replace only a small part of the external volume. In this work we have compared the release results obtained from both methods.

Concretely, in our release dialysis method, we used the non-commercial DOX and 5FU. A volume of 2.5 mL of drug–microgel complex buffered solution (pH 7) was added to a dialysis tube (Pur-A-Lyzer Maxi 12,000 Da), which was inserted into a tube containing 15 mL of buffered solution. This tube was placed in the thermal shaker (Thermal Shaker Heat/Cool, Ohaus) at 37 °C and 500 rpm. At specific intervals (15, 30, 45, 60, 90, 120, 300, 510, 1380, 1500, 1620 min), an external volume was removed to measure the corresponding released drug concentration and was replaced by the same volume of fresh buffer solution. The same experiment was made using 2.5 mL of free-drug (or microgel) buffered solution as the internal volume to carry out the blanks. This removed external volume was 15 mL (all the external volume) or only 0.5 mL.

The removed drug concentration can be analyzed by UV-Vis spectrophotometry or by ultra-high-performance liquid chromatography (UHPLC). In this work, we compared the results from both techniques using a UV-Vis Eppendorf BioSpectrometer (Hamburg, Germany) and a Waters Xevo Tq-S mass spectrometer (Milford, MA, USA) (Centre for Scientific Instrumentation of the University of Granada), which is a more appropriate technique for diluted drug solutions. Considering the preliminary results, for the encapsulation protocol a calibration was performed in MilliQ water, while for the release protocol the calibration was performed in buffer of pH 7 and at lower drug concentrations. This is due to the lower absorbance signal detected in the release experiments when compared with the encapsulation experiments. Thus, the cumulative mass of drug removed at each measurement time was determined and related to the drug encapsulated mass. All experiments were conducted in triplicate.

## Figures and Tables

**Table 1:** Characterization by static and dynamic light scattering of microgel particles in Milli-Q water. The first columns list the particle radius obtained from the RGD model (R_RGD_, Equation (1)), followed by the fuzzy-sphere parameters R and σ_fuzzy_ (Equation (2)) and the corresponding effective static radius R_SLS_ = R + 2σ_fuzzy_. The last two columns report the hydrodynamic radius determined by the cumulants method (R_h_^cum^, Equation (4)) and by the convection-corrected model (R_h_^conv^, Equation (5)), respectively.

	SLS				DLS	
	RGD Model	Fuzzy-Sphere Model			Cumulant	Convection
T (°C)	R_RGD_ (nm)	R (nm)	σ_fuzzy_ (nm)	R_SLS_ (nm)	R_h_^cum^ (nm)	R_h_^conv^ (nm)
25	263 ± 2	267 ± 3	56 ± 3	379 ± 5	520 ± 70	530 ± 60
43	209.1 ± 0.4	209.1 ± 0.4	0	209.1 ± 0.4	221 ± 15	265 ± 19

**Table 2:** Evolution with time of the mean and mode values of the hydrodynamic diameter of the microgel measured by NTA in different media to 43 °C. Errors of the mean correspond to the standard deviation (SD) of the size distribution.

	Initial Time (t = 0)		After 40 min	
Medium	Mean Diameter (nm)	Mode (nm)	Mean Diameter (nm)	Mode (nm)
Buffer pH 4	420 ± 150	460	430 ± 130	490
Milli-Q water	480 ± 150	495	600 ± 130	620
Buffer pH 7	660 ± 130	715	580 ± 150	600

**Table 3:** Static and dynamic characterization of PNIPAM microgels at 43 °C in buffer (pH 4), Milli-Q water and buffer (pH 7). The first columns list the static radii obtained from SLS by fitting the Rayleigh–Gans–Debye model (R_RGD_) and the fuzzy-sphere model (R, σ_fuzzy_), together with the corresponding effective static radius R_SLS_ = R + 2σ_fuzzy_. The last two columns report the hydrodynamic radii determined from 3D-DLS using the cumulants method (R_h_^cum^) and the convection-corrected model (R_h_^conv^). Uncertainties correspond to standard deviations from at least three independent measurements.

	SLS				DLS	
	RGD Model	Fuzzy-Sphere Model			Cumulant	Convection
Medium	R_RGD_ (nm)	R (nm)	σ_fuzzy_ (nm)	R_SLS_ (nm)	R_h_^cum^ (nm)	R_h_^conv^ (nm)
Buffer pH4	220.3 ± 0.5	220.3 ± 0.5	0	220.3 ± 0.5	396 ± 7	367 ± 9
Milli Q water	209.1 ± 0.4	209.1 ± 0.4	0	209.1 ± 0.4	221 ± 15	265 ± 19
Buffer pH7	213.0 ± 0.5	212.0 ± 0.6	1.0 ± 0.2	214.0 ± 0.7	375 ± 10	362 ± 6

## Data Availability

The data presented in this study are available on request from the corresponding authors.

## Abbreviations

The following abbreviations are used in this manuscript:

PNIPAM	Poly(N-isopropylacrylamide)
NPs	Nanoparticles
VPTT	Volume phase transition temperature
DLS	Dynamic light scattering
DOX	Doxorubicin
5FU	5-Fluorouracil
BCS	Biopharmaceutics classification system
TEM	Transmission electron microscopy
AFM	Atomic force microscopy
SLS	Static light scattering
NTA	Nanoparticle tracking analysis
LDE	Laser Doppler Electrophoresis
RGD	Rayleigh-Gans-Debye
3-BA	3-Butenoic acid
EE	Encapsulation efficiency
DL	Drug loading
BIS	methylenebisacrylamide
V50	2,2′-azobis(2-methylpropionamidine)
HPLC	High performance liquid chromatography
UHPLC	Ultra high performance liquid chromatography
Abs^max^	Maximum absorbance
M_I_	Mass of added drug
M_F_	Free non-encapsulated drug

## Appendix A

### Standard DLS Characterization

From the analysis of the size distribution by intensity of the microgel in Milli-Q water (see Table A1), we obtained mean diameters in the swollen state that were higher than expected, and with wide standard deviations. Around the VPTT of PNIPAM, we only got valid results at 36 °C and 39 °C. In the collapsed state, diameters were in the range predicted by AFM, and not far from those obtained by NTA.

**Table A1:** Mean diameter of microgel in Milli-Q water at different temperatures from the analysis of the size distribution by intensity (DLS, capillary cell).

T (°C)	Mean Diameter (nm)
18	1850 ± 340
21	2900 ± 1200
24	2800 ± 900
36	1250 ± 230
39	1170 ± 170
42	970 ± 200
45	900 ± 300
48	870 ± 180

**Table A2:** Electrophoretic mobility of microgel system at T = 43 °C and in different media.

Medium	Electrophoretic Mobility (10^−8^ m^2^/Vs)
Milli-Q water	−2.3 ± 0.4
Buffer pH 4	1.7 ± 0.4
Buffer pH 7	−2.2 ± 0.4
